# Interpreting a Sudden Population Decline in a Long‐Lived Species ( *Malaclemys terrapin rhizophorarum* )

**DOI:** 10.1002/ece3.71347

**Published:** 2025-05-07

**Authors:** Jacquelyn C. Guzy, Brian J. Smith, Mathew J. Denton, Michael S. Cherkiss, David C. Roche, Andrew G. Crowder, Kristen M. Hart

**Affiliations:** ^1^ U.S. Geological Survey Wetland and Aquatic Research Center Davie Florida USA; ^2^ Department of Wildland Resources and Ecology Center Utah State University Logan Utah USA; ^3^ U.S. Geological Survey Wetland and Aquatic Research Center Gainesville Florida USA; ^4^ Xylem Analytics Boca Raton Florida USA

**Keywords:** demography, diamond‐backed terrapin, emigration, Everglades National Park, Florida, illegal harvest, mangrove, poaching, population model, survival

## Abstract

Long‐term ecological studies are critical for providing insight into population dynamics and detecting population declines, particularly for species of conservation concern. However, spatiotemporal variation and logistical challenges make the identification of sudden population declines difficult. We conducted an in‐water capture‐mark‐recapture study of mangrove diamond‐backed terrapins ( *Malaclemys terrapin rhizophorarum* ) within Big Sable Creek, in Everglades National Park, Florida. We used an 18‐year dataset (2001 to 2019) incorporating year, sex, hurricane occurrence, and sampling effort to estimate survival using Cormack–Jolly–Seber (CJS) models in Program Mark. Annual survivorship estimates were high from 2001 to 2003 for both sexes (91%–96%) and variable from 2006 to 2014 (77%–92%). Beginning in 2015, survival estimates exhibited a steeper decline (females: 65%, males 75%), and dropped to below 36% by 2018. Because the driver of this apparent population decline is unknown, we created a population projection matrix and used model‐estimated annual survival to simulate annual terrapin population size. We then generated competing scenarios of low survival at various age classes to attempt to reproduce a simulated decline mirroring what we observed from our capture data. A scenario of low adult survival (75%–85%) from 2012 to 2018, possibly in conjunction with no reproduction after 2010, provides estimates of abundance that appear to match simulated annual population size and may indicate that adult emigration/human removal or a drastic drop in recruitment could be responsible for the apparent decline in survival. We explore reasons for this apparent decline and highlight difficulties common to long‐term studies that may influence how declines are interpreted.

## Graphical abstract

Annual sex‐specific survival estimates for mangrove diamond‐backed terrapins ( *Malaclemys terrapin rhizophorarum* ) in Everglades National Park from 2001 to 2019.

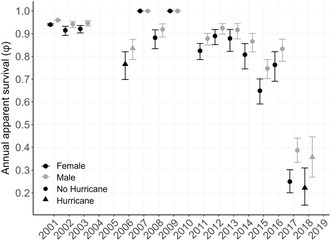

## 1 Introduction

An important component of conservation science is monitoring trends of vulnerable species to determine which populations are declining and why (Fournier et al. 2019; White 2019). Detecting and measuring population declines is challenging because understanding population dynamics can require a long time series to incorporate measurement uncertainty and account for natural variation, random fluctuations, environmental change, and ecological time lags; as a result, these studies are often labor intensive and expensive (Meyer et al. 2010; McCain et al. 2016; Ost et al. 2016; Didham et al. 2020; Watts et al. 2020). Long‐term studies (generally > 10 years) are important for quantifying the complex interactions prevalent in many ecosystems and these studies can identify trends and shifting baselines, document uncommon events, and capture unanticipated ecological surprises (Magnuson 1990; Lovett et al. 2007; Doak et al. 2008; Lindenmayer et al. 2010; Hughes et al. 2017).

The southeastern United States is a well‐known global hotspot of turtle biodiversity (Buhlmann et al. 2009). Yet, turtles and tortoises are among the most imperiled vertebrates on the planet, more so than birds, mammals, fishes, or even amphibians (Lovich, Ennen, et al. 2018; Rhodin et al. 2018; Stanford et al. 2020), and more than half of species are listed as critically endangered, endangered, or vulnerable (IUCN Red List; Rhodin et al. 2018). Turtles have important roles in terrestrial and aquatic ecosystems, from seed dispersal to mineral cycling and carbon storage (Lovich, Ennen, et al. 2018). The primary threats facing turtles and tortoises are habitat loss (i.e., fragmentation, degradation, deforestation, urbanization, agricultural conversion), urbanization (i.e., road mortality, nest and hatchling depredation by subsidized predators, environmental pollution, invasive species), climate change, and overcollection of turtles and their eggs (i.e., food consumption, international pet trade, and traditional medicines; reviewed in Rhodin et al. 2018; Stanford et al. 2020). These impacts decrease the number and size of populations, and consequently, dramatically reduce the important ecological roles that turtles and tortoises normally fulfill in their habitats and ecosystems (Lovich, Ennen, et al. 2018). The geographic hotspots for turtle declines are in Southeastern China, Southeast Asia, and Indonesia, Northern India and Pakistan, the Amazon region of South America, Africa, and North America, especially in the Northeastern United States (Rhodin et al. 2018). Given the general life‐history characteristics of many turtles, including delayed sexual maturity, longevity, terrestrial nesting activity, and lack of parental care, they are particularly vulnerable to human‐induced threats (Congdon et al. 1993; Doak et al. 1994; Heppell 1998; Heppell et al. 1999). Despite iteroparous reproduction, turtles recover slowly from declines because their populations require high juvenile and adult survival for persistence (Congdon et al. 1993; Heppell 1998). Thus, increased mortality in the juvenile or adult stages can cause population declines (Heppell et al. 1996; Heppell 1998).

The diamond‐backed terrapin ( *Malaclemys terrapin* ) inhabits brackish waters within coastal salt marsh and mangrove habitats of Bermuda and along the Atlantic and Gulf Coasts of the United States from Cape Cod, Massachusetts to southern Texas (Roosenburg and Kennedy 2018). The species has been designated as vulnerable by the International Union for Conservation of Nature (IUCN; Roosenburg et al. 2019) because of several main threats: (1) mortality of juveniles and adults as bycatch in crab pots (reviewed in Chambers and Maerz 2018) and of nesting females on roads (reviewed in Maerz et al. 2018), (2) loss of nesting and juvenile habitat to development and sea level rise, (3) the effect of subsidized predators at nesting beaches, and (4) loss of estuarine saltmarsh and mangrove habitats throughout their range (reviewed in Maerz et al. 2018). However, an additional but not well‐documented threat is from commercial and illegal harvesting and overcollection for the pet trade, which is one focus of this study. Diamond‐backed terrapins have sustained range‐wide population declines of 25%–30% over the last 30 years, and that rate of decline is predicted to continue, amounting to an additional 35%–45% decline over three generations (45 years; Roosenburg et al. 2019). In a survey of 54 researchers from 16 states where terrapins occur, 30% indicated populations were declining in their state, 15% indicated populations were stable, and 55% indicated unknown status (Butler et al. 2006).

Uncertainty exists regarding the status and trends in terrapin populations. Despite decades of research described in Roosenburg and Kennedy (2018), significant gaps remain in the basic natural history of terrapin populations throughout their range, particularly in eastern Louisiana, Pamlico Sound, North Carolina, and along the Florida Gulf Coast (Lovich, Whitfield Gibbons, et al. 2018). More specifically, despite their extensive range, long‐term demographic studies of diamond‐backed terrapins spanning several decades only exist for four locations, all on the Atlantic Coast (Kiawah Island, SC; Cape May Peninsula, NJ; Mechanicsville, MD; and Cape Canaveral, FL, e.g., Montevecchi and Burger 1975; Wood 1997; Seigel et al. 2002; Dorcas and Gibbons 2013, reviewed by Lovich, Whitfield Gibbons, et al. 2018). Yet, the successful implementation of conservation and management strategies depends in part on a solid understanding of geographic variation in life history traits and how it affects population demographics. Historically, seven subspecies of diamond‐backed terrapin have been recognized based on morphological characteristics (Carr 1952; Converse and Kuchta 2018), although more recent work suggests there are four genetically distinct lineages (Hart et al. 2014), with Florida populations strongly differentiated from neighboring populations in the Atlantic and Gulf (Hauswaldt and Glenn 2005; Drabeck et al. 2014; Hart et al. 2014). The focal taxon in this study is the mangrove diamond‐backed terrapin subspecies, *M. t. rhizophorarum*, which is restricted to southern Florida (Lovich and Hart 2018) and occupies creeks within dense mangroves as compared to salt marsh creeks typical of terrapins elsewhere throughout their range. There is little demographic information (but see Hart and McIvor 2008), and no data on survival rates for this subspecies.

Because anthropogenic threats to terrapins continue, with population pressure mounting in all coastal environments, and future survival tenuous in some locales (Butler and Roosenburg 2018), we initially sought to establish baseline demographic and abundance estimates for mangrove terrapins in a remote, largely unimpacted location. However, as the study progressed over two decades, we documented an unexpected and sudden apparent decline in this population of terrapins. Causes for the decline are unknown but herein we explore several hypotheses and potential mechanisms (Table 1).

**TABLE 1:** Hypotheses, potential mechanisms, and analytical approach used to interpret a sudden population decline in a population of mangrove diamond‐backed terrapin ( *Malaclemys terrapin rhizophorarum* ) in Everglades National Park, Florida, USA.

Observation	Hypothesis	Potential mechanism	Expected outcome	Does evidence support mechanism	Analytical approach	Supplemental file (S1) panel
Declining captures	Reduction in adult survival	Permanent emigration	Relocation to a new area; systematic decrease in survival each year	Unlikely	Simulation	H, I, J, K, L
			Human removal; systematic decrease in survival each year	Likely		H, I, J, K, L
			Hurricane; sharp decrease in survival post hurricane	No		E
		Natural mortality	Slow rate of declining captures	No	Observation	
		Disease	Signs of illness, infection, death	No		
		Bycatch	Trap presence, deaths	No		
		Increased predation	Predator population increase	No		
		Water quality	Increased turbidity, salinity changes	No		
		Prey availability	Reduction in body condition index	No		
	Reduced reproduction	Nesting location change	No reproduction	Possible	Simulation	G, K, L
		Low adult survival 2010	Sharp drop in survival in 2014, given 4‐year time lag for hatchlings to mature and recruit to study site	No		F
		Hurricane alters nesting beach	Reduced reproduction	No		C
	Reduced recruitment	Increased predation	Predator population increase	No	Observation	
		Hurricane alters nesting beach	Reduced reproduction	No	Simulation	B
	Reduction in juvenile survival	Hurricane	Death of hatchlings or juveniles	No	Simulation	D
		Juvenile habitat location change	Juveniles do not recruit into study site	No		K, L

## 2 Methods

### 2.1 Study Site

We sampled terrapins from a forested mangrove creek system within Big Sable Creek, at Cape Sable, along the western portion of Everglades National Park (ENP) in southwestern Florida. This creek system is a mosaic of mangrove forest overstory ( *Rhizophora mangle* and *Avicennia germinans* ) and intertidal mudflats exposed during daily tidal fluctuations of up to 1.2 m. Mangrove creeks in this system are open‐canopy narrow (~10–20 m wide), shallow (~1–4 m deep), and although tannic in some areas, the water is clear, particularly during outgoing tides. The study area is a remote backcountry wilderness accessible only by water, and the nearest road or urban development is 30 km away.

### 2.2 Data Collection

We conducted terrapin surveys from 2001 to 2019, between November and January annually, when both sexes occupy the creek system (Hart 2005). Surveys took place over an average of 4.26 days each year. In some years, 1–3 additional surveys occurred between February and October (2002, 2003, 2006, 2008, 2009, 2012, 2017), and in 2 years, sampling did not take place (2004 and 2005). Surveys were conducted from small boats during falling and rising tides. Terrapins were highly visible throughout the water column and were captured using dip nets. For each individual, photographs were taken, and morphometric measurements were recorded (i.e., mass, carapace width, and straight and curved carapace length [SCL, CCL] and plastron length [SPL, CPL]). New turtles were given a unique passive integrated transponder (PIT) tag (Biomark, Boise, ID, 8 mm tag;Buhlmann and Tuberville 1998) and individually marked by notching the marginal scutes in a systematic pattern (e.g., Cagle 1939). All turtles were released after workup in the creek they were captured in. In 2020–2022, additional surveys were conducted to further establish apparent population trends; however, these data were not included in survival or population projection analyses.

### 2.3 Data Analysis

#### 2.3.1 Body Size Relationships

We examined body size relationships by removing outliers representing obvious measurement error, and plotted length versus mass. Because terrapin captures began to decline in 2014, we compared body size for all years prior to and including 2014 (i.e., years pooled; ‘Before 2014’ category) with data from years after 2014 (i.e., years pooled; ‘After 2014’ category), using an analysis of covariance (ANCOVA; log(mass) ~ plastron length + year_category). An ANCOVA was used to control for inherent effect of length on mass as an index of body condition. Model assumptions were examined and verified using residual plots (i.e., density, Q–Q, and fitted vs. residual plots). Body size analyses were performed using package ‘stats’ in R version 4.2.2 (R Core Team 2023).

#### 2.3.2 Survival Estimation

To estimate terrapin apparent survival, we created individual capture histories for each turtle and fit a Cormack–Jolly–Seber (CJS) survival model (Cormack 1964; Jolly 1965; Seber 1965) using Program Mark via the RMark package (Laake 2013; Laake and Rexstad 2022) in R version 4.2.1 (R Core Team 2023). We incorporated variables that we expected to influence terrapin survival $\left(\phi \right)$, including sex, year, and whether a hurricane had passed through the site in a given year. We also included variables expected to influence capture probability, including sex and effort (i.e., number of hours per survey). Therefore, the model formulation we used was: ϕ(sex, hurricane, year), p(sex, effort). Body size was not incorporated as terrapins in this study are predominantly sexually mature adults (females 96%, males 97%) as defined by Siegel 1984 (i.e., females > 13.5 cm SPL, males > 9.5 cm SPL). Note that this also implies our survival estimates are only for the adult age class (see below).

#### 2.3.3 Simulated Population Size

**FIGURE 1:**
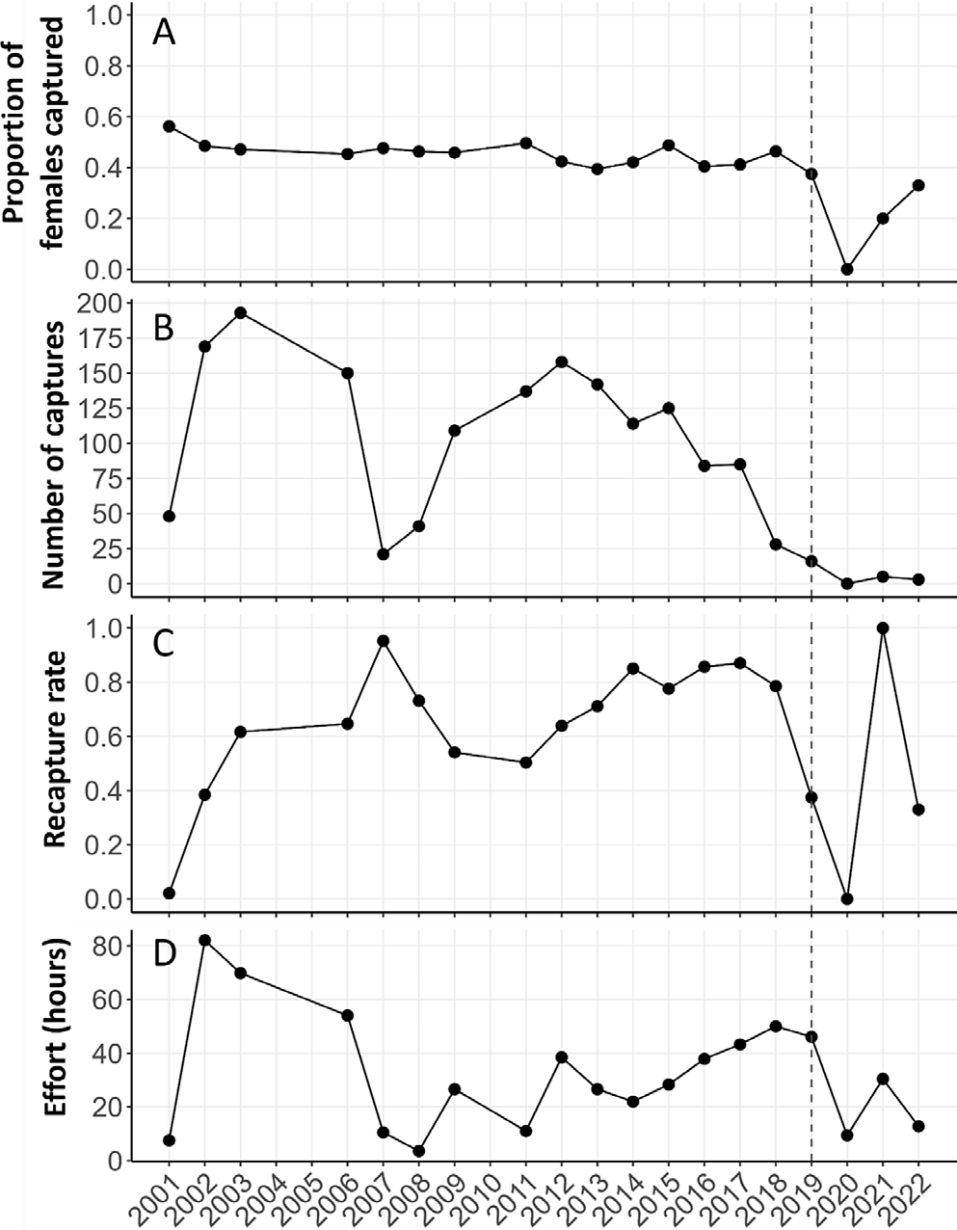
Mangrove diamond‐backed terrapin ( *Malaclemys terrapin rhizophorarum* ) captures in Everglades National Park, Florida, USA between 2001 and 2022. Panel (A) proportion of females captured each year, (B) number of individuals captured each year, (C) annual recapture rates, and (D) annual sampling effort (h). Surveys were not conducted in 2004 and 2005. Data to the right of the dashed vertical line were from informal surveys not included in analyses but intended data to further establish apparent population trends.

Because we only observed a portion of this structured population (the mature adults), we also used simulation to explore hypotheses for declines in captures over time that may have been caused by effects operating on other ages. We modified the Leslie matrix population model of Mitro (2003) by incorporating adult female annual survival estimates from this study, along with other age class values provided in Mitro (2003), and life‐history information from the literature to simulate annual terrapin population size for each year of the study. Specifically, as in Mitro (2003), we constructed and parameterized an age‐based, female‐only matrix population model for diamond‐backed terrapins in our study area: **n**(*t* + 1) = **An**(*t*) where **n**(*t*) is a vector of age‐specific abundances at year t and **A** is the population projection matrix. We will refer to each element of **n**(*t*) as *n*_*i*_(*t*). Elements of matrix **A** describe survival and reproduction transitions among ages between years. We chose to model only the female portion of the population because our observed sex ratios were nearly constant throughout the study (Figure 1A). The reproductive output or fertility (F) for a female diamond‐backed terrapin is the number of female offspring produced per female in 1 year that survived to the next (Caswell 2001). The number of female hatchlings per breeding female diamond‐backed terrapin is a combination of the number of eggs per clutch, the number of clutches per season, the probability of laying a successful clutch, the probability of eggs in a successful clutch hatching, and the proportion of female offspring (Mitro 2003). For several of these variables, data are sparse, particularly for Florida terrapins. We used a clutch size of 6.7 based on data from Seigel (1980) who studied terrapins on the east coast of Florida and documented that the clutch size of *M. terrapin tequesta* is considerably smaller than that of northern populations. Studies indicate that the number of clutches laid per season for terrapins is likely 2 or 3 (Seigel 1980; Ernst and Lovich 2009; Lovich, Whitfield Gibbons, et al. 2018), and others have also noted the occurrence of multiple clutches (Roosenburg and Dunham 1997; Feinberg and Burke 2003; Donini et al. 2018). However, because Florida has the longest nesting (78 days; Butler et al. 2004) and activity seasons (mid‐February to late November; Seigel 1984), and Florida terrapins have smaller clutch sizes (6.7 eggs; Seigel 1980) compared to the northeastern United States (15 eggs; Mitro 2003), we expected the overall number of clutches laid for terrapins at the extreme southern portion of their range, where our study occurs, to be higher. Therefore, we used a value of 2.5 clutches per season. We used values provided by Mitro (2003) for probability of clutch survival (0.097) and egg survival (0.8735). The sex ratio for our study is approximately 1:1 and thus we used a value of 0.5 for proportion of female offspring (Figure 1A). Combining these values resulted in a value of 0.7096 female hatchlings per breeding female diamond‐backed terrapin, and this value was multiplied by the juvenile survival rate (see below) for the remaining 9 months of the year S_j_^(9/12)^, to yield an estimate of F. Age at first breeding for female diamond‐backed terrapin varies geographically (Seigel 1984; Lovich and Gibbons 1990; Roosenburg 1990) but data from Florida indicate most are mature by age 4 (Seigel 1984). Therefore, all diamond‐backed terrapins of reproductive age (age 4 and older) were grouped into a final stage in the model. Following Mitro (2003) we assumed a juvenile survival rate *S*_j_ for ages 0–2 and a subadult survival rate *S*_sa_ equal to adult survival rate *S*_a_ for subadult age 3 (i.e., *S*_sa_ = *S*_a_). The adult survival rate *P*_a_ (see Mitro 2003) for combined ages 4+ was a function of annual adult survival *S*_a_, age at first breeding m, and longevity *l*: where *T* is the stage duration (*T* = l−*m* + 1) and *λ* = 1 (Caswell 2001), and longevity was set as 40 years (Hildebrand 1932).

$$P_{a}=S_{a}\left(1-\frac{{\left(\frac{S_{a}}{\lambda }\right)}^{T}-{\left(\frac{S_{a}}{\lambda }\right)}^{T-1}}{{\left(\frac{S_{a}}{\lambda }\right)}^{T}-1}\right)$$

We used the adult apparent female survival rate estimated for each year of our study generated from our mark–recapture dataset. There are no available estimates of juvenile survival (*S*_j_) rate for the diamond‐backed terrapin available in the literature, and this parameter was the only unknown or uninformed parameter in the projection matrix. Therefore, Mitro (2003) estimated *S*_j_ by setting projected *λ* equal to realized *λ* (from mark–recapture data) and solved for *S*_j_ (*λ* = 1.034; *S*_j_ = 0.565). The resulting projection matrix ***A*** for a diamond‐backed terrapin population with age at first breeding at 4 years was:

$$A=\left[\begin{array}{ccccc} 0 & 0 & 0 & 0 & F\\ S_{j} & 0 & 0 & 0 & 0\\ 0 & S_{j} & 0 & 0 & 0\\ 0 & 0 & S_{\mathrm{sa}} & 0 & 0\\ 0 & 0 & 0 & S_{\mathrm{sa}} & P_{a} \end{array}\right]$$

The stable age distribution for the diamond‐backed terrapin population was equal to the right eigenvector of projection matrix ***A*** (above) which we calculated using the “popbio” package in R version 4.2.1 (Stubben and Milligan 2007; R Core Team 2023). We set an initial total population size of 500, a value larger than the highest number of captures in a given year. We distributed these 500 individuals among the age classes assuming the stable age distribution. Using the demographic parameters described above, in conjunction with annual survival estimates from mark‐recapture surveys (see Section 2.3.2), we simulated the population size for each year by multiplying the year‐specific matrix by the population size in the previous time step. We incorporated demographic stochasticity by sampling the number of surviving individuals from a binomial distribution (i.e., the sum of binary outcomes) and number of recruits from a Poisson distribution, as implied by the Leslie matrix. That is, we modeled the number of individuals in age class *i* at time *t*, *n*_*i*_(*t*) as random variables as follows:

$$n_{0}\left(t\right)∼\text{Poisson}\left(\lambda =n_{4+}\left(t\right)\times F\right)$$

$$i\in \left\{1:3\right\};n_{i}\left(t\right)∼\text{Binomial}\left(N=n_{i-1}\left(t-1\right),p=S_{i}\right)$$

$$\begin{array}{c} n_{4+}\left(t\right)∼\text{Binomial}\left(N=n_{3}\left(t-1\right),p=S_{\mathrm{sa}}\right)\\ +\text{Binomial}\left(N=n_{4+}\left(t-1\right),p=P_{a}\right). \end{array}$$

We chose the binomial distribution for survival and the Poisson distribution for fecundity as the most parsimonious distributions to represent the process, rather than assuming some form of overdispersion. We ran these simulations for each year, 10,000 times. We then summed the number of individuals in each stage to estimate the total adult female population size each year (i.e., proxy for annual abundance). Refer to Guzy et al. (2025) for code used to simulate population size.

#### 2.3.4 Population Decline Simulation

To reproduce a decline similar to what we observed in our capture data and in the pattern of simulated annual abundance, we generated competing scenarios of low survival and/or recruitment in several age classes to attempt to identify potential causes for the decline. For example, we simulated low survival during the 2 years where hurricanes passed close to the study site [Wilma in 2005 (Pasch et al. 2006) and Irma in 2017 (Cangialosi et al. 2021)] during these years nests may have flooded or hatchlings may have died or been displaced from the study area. We also simulated low adult survival in 2010 because capture numbers began to decline in 2014, and thus 2010 accounts for the approximate 4‐year time lag it takes for turtles to mature and potentially recruit into our study system (i.e., minimal reproduction in 2010). We also considered scenarios of very low adult survival during different years because of emigration out of the study system, death, or removal of turtles by humans (i.e., illegal harvest). In total, we simulated annual abundance under 11 scenarios (Figure S1), including no hatchling survival during hurricane years, no fecundity during hurricane years, no survival for young age classes during hurricane years, 50% adult survival after a hurricane year, 50% adult survival in 2010, no reproduction after 2010, adult survival 60% from 2012 to 2018, adult survival 85% from 2012 to 2018, adult survival 75% from 2012 to 2018, no reproduction after 2010 and 75% adult survival from 2012 to 2018, and no reproduction after 2010 with 85% adult survival from 2012 to 2018. Refer to Guzy et al. (2025) for code used to execute population decline simulations in R version 4.2.1 (R Core Team 2023).

## 3 Results

### 3.1 Captures

Between 2001 and 2019, we captured 656 individual *M. terrapin* (1789 total captures). Sex ratios were approximately 1:1 (female: *n* = 333; male: *n* = 323; Figure 1A) and all individuals were adults. Over this time, we conducted 98 surveys (mean 4.26 surveys per year) totaling 558 survey hours (mean 5.7 h per survey). The number of captures per survey varied with effort but was relatively high at an average of 117 captures per year across 13 years (2001 through 2014; Figure 1B). Thereafter, the number of captures steadily declined, dropping to an average of 68 from 2016 through 2019, despite increasing effort in the study site (Figure 1D). Return visits to the site with a moderate effort of between 9.41 and 30.4 search hours in 2020, 2021, and 2022 resulted in 0, 5, and 3 individuals captured, respectively (Figure 1B). After initial surveys in 2001, recapture rates remained high (mean 68.2%) and were relatively consistent throughout the study until 2020 (Figure 1C).

### 3.2 Body Size

**FIGURE 2:**
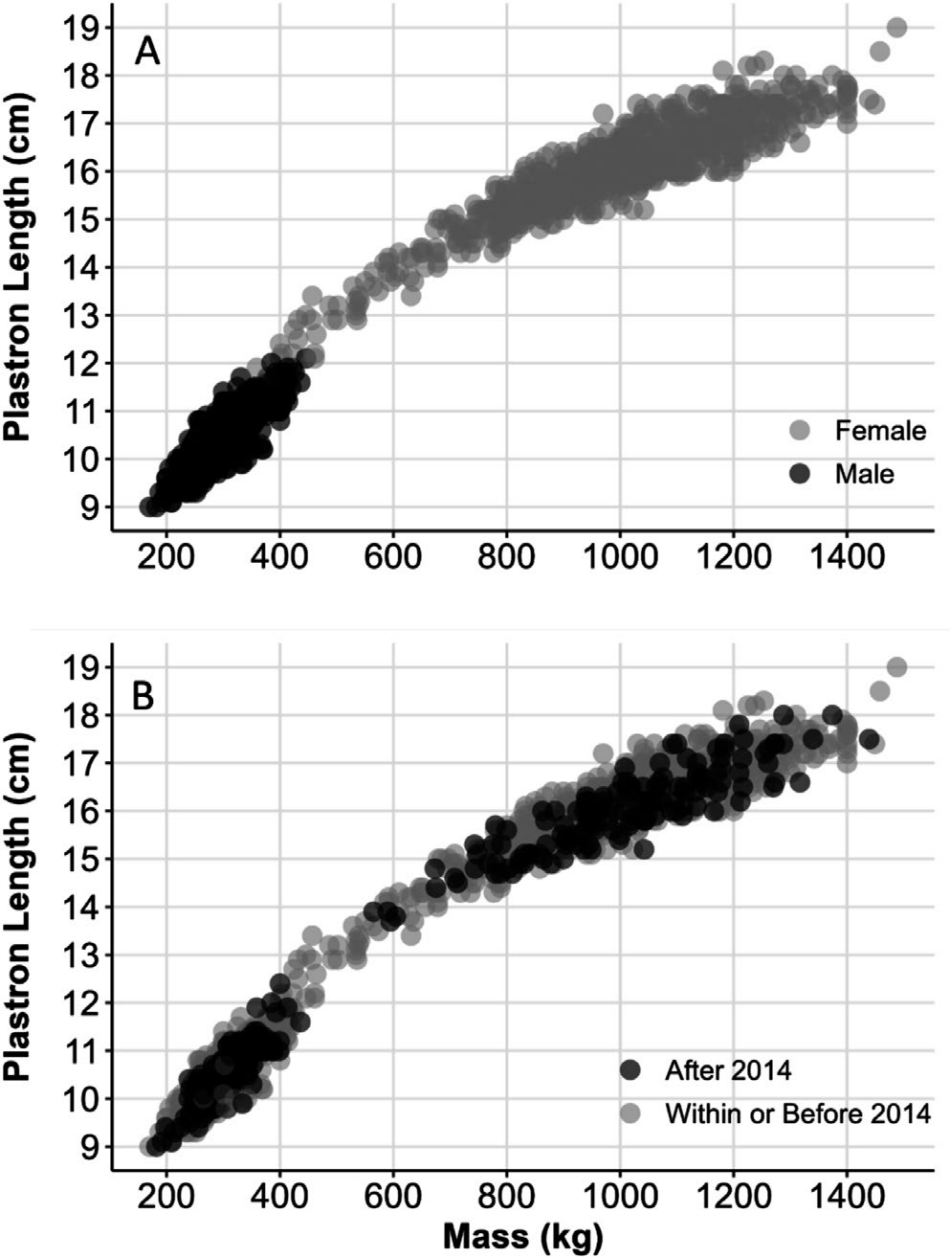
Mangrove diamond‐backed terrapin ( *Malaclemys terrapin rhizophorarum* ) body size in Everglades National Park, Florida, USA between 2001 and 2022. Panel (A) Relationship between plastron length and mass (males, *n* = 988 captures; females, *n* = 793 captures). Panel (B) Body size before (*n* = 1441 captures) and after (*n* = 340 captures) an apparent population decline began circa 2014.

Morphometric measurements illustrate terrapin sexual size dimorphism, with males smaller than females (i.e., adult male plastron length 9.0 to 12.1 cm and mass 140–446 g; adult female plastron length 10.1–19.0 cm and mass 254–1488 g; Figure 2A). The relationship between mass and plastron length did not vary by survey time period (ANCOVA slope of time period term, *p* = 0.487; ANCOVA slope of interaction of length and time period term, *p* = 0.766; Figure 2B).

### 3.3 Apparent Survival

**FIGURE 3:**
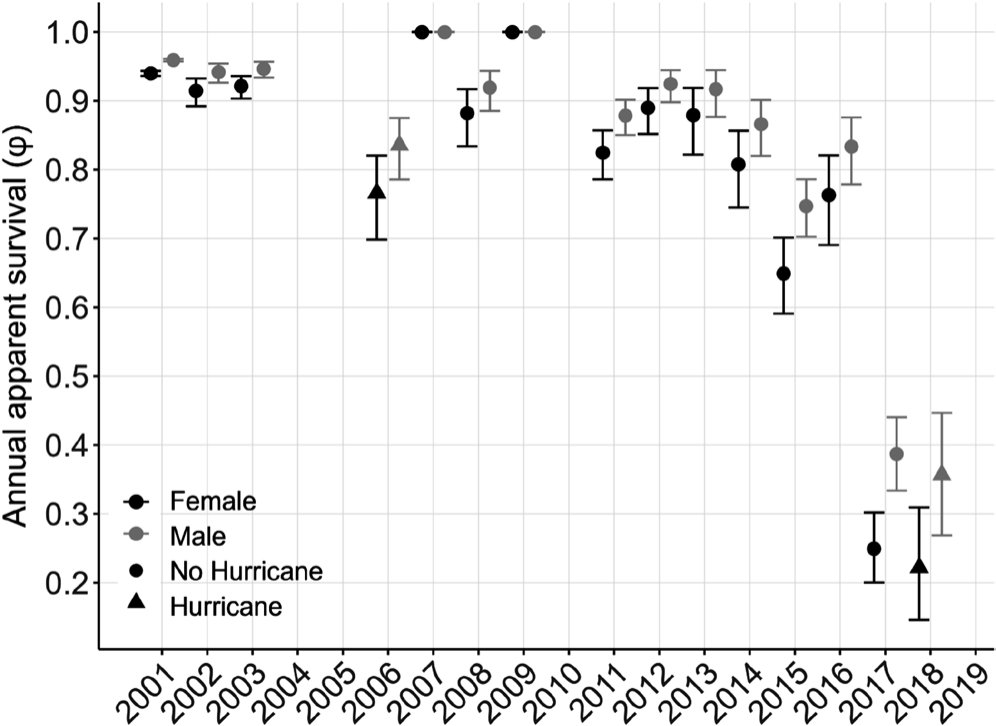
Annual sex‐specific survival estimates for mangrove diamond‐backed terrapins ( *Malaclemys terrapin rhizophorarum* ) in Everglades National Park (females = black circles; males = gray circles; triangles = hurricane years) from 2001 to 2019. Bars represent 95% confidence intervals. Survival inestimable for 2007 and 2009 due to low sample size.

Annual survival estimates were high from 2001 to 2003 for both sexes (91%–96%) and variable from 2006 to 2014 (77%–92%; Figure 3). Survival began to decline in 2006 (females: 77%, males: 84%), and estimates were variable through 2014 (81%–92%), with unreliable estimates in 2007 and 2009 due to small sample sizes in those years (i.e., low effort and few captures; Figure 1B–D). In 2015 and 2016, estimates dropped to 65%–76% (95% CI 60%–82%) for females and 75%–83% (95% CI 70%–88%) for males. By 2018, apparent survival dropped to 22% (95% CI 15%–31%) for females and 36% (95% CI 27%–45%) for males. Survival estimates were marginally lower during hurricane years, but the effect was weak (i.e., confidence intervals overlapped estimates for neighboring years; Figure 3), and hurricane population simulations (Figure S1) were not similar to the pattern of decline we observed in capture numbers (Figure 1B).

### 3.4 Capture Probability

**FIGURE 4:**
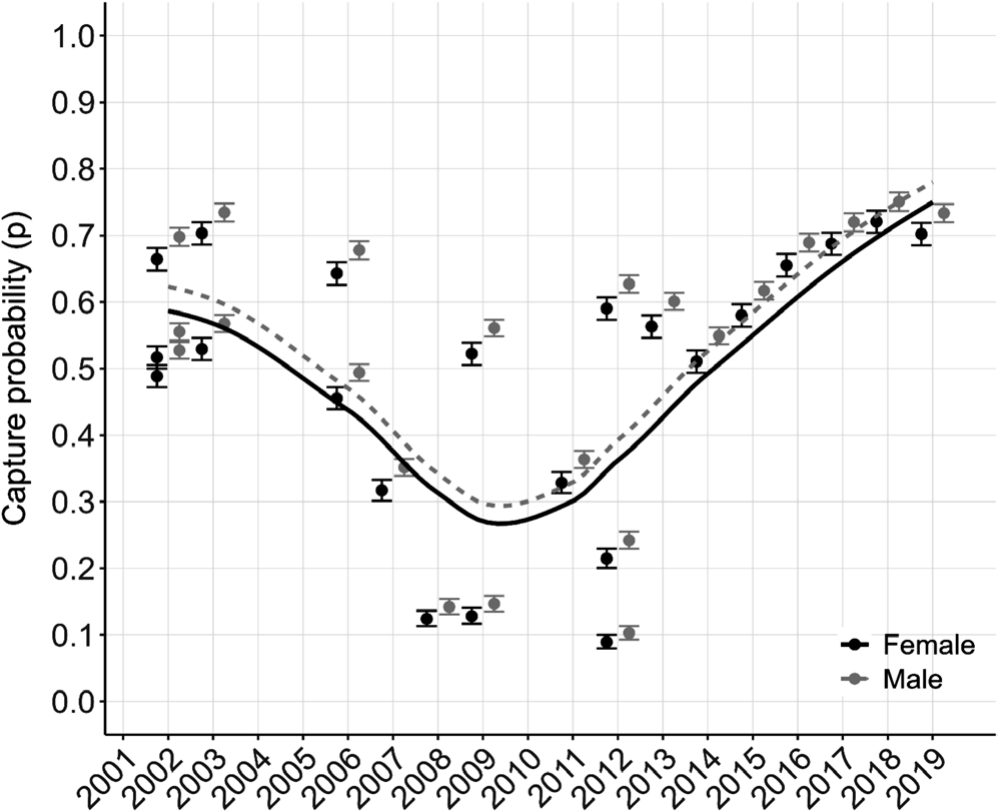
Annual sex‐specific capture probability of mangrove diamond‐backed terrapins ( *Malaclemys terrapin rhizophorarum* ) in Everglades National Park from 2001 to 2019 (females = black circles; males = gray circles). Estimates incorporate effort (i.e., the number of hours per survey) which increased in later years. Bars represent 95% confidence intervals.

Capture probability (i.e., detection probability) was similar among sexes (males: 10%–75%; females: 9%–72%; Figure 4), was generally high (i.e., > 40%), and varied by year. Recapture rates increased with effort, as expected (Figures 1C and 4). Specifically, between 2007 and 2014, sampling effort was lower and more variable, ranging from 3.58 to 38.5 survey hours (Figure 1C); during this time capture probability varied from 9% to 60% (Figure 4). Between 2015 and 2019, sampling effort increased, varying from 28.3 to 50 survey hours (Figure 1C) and capture probability varied from 58% to 75% (Figure 4).

### 3.5 Simulated Population Size

**FIGURE 5:**
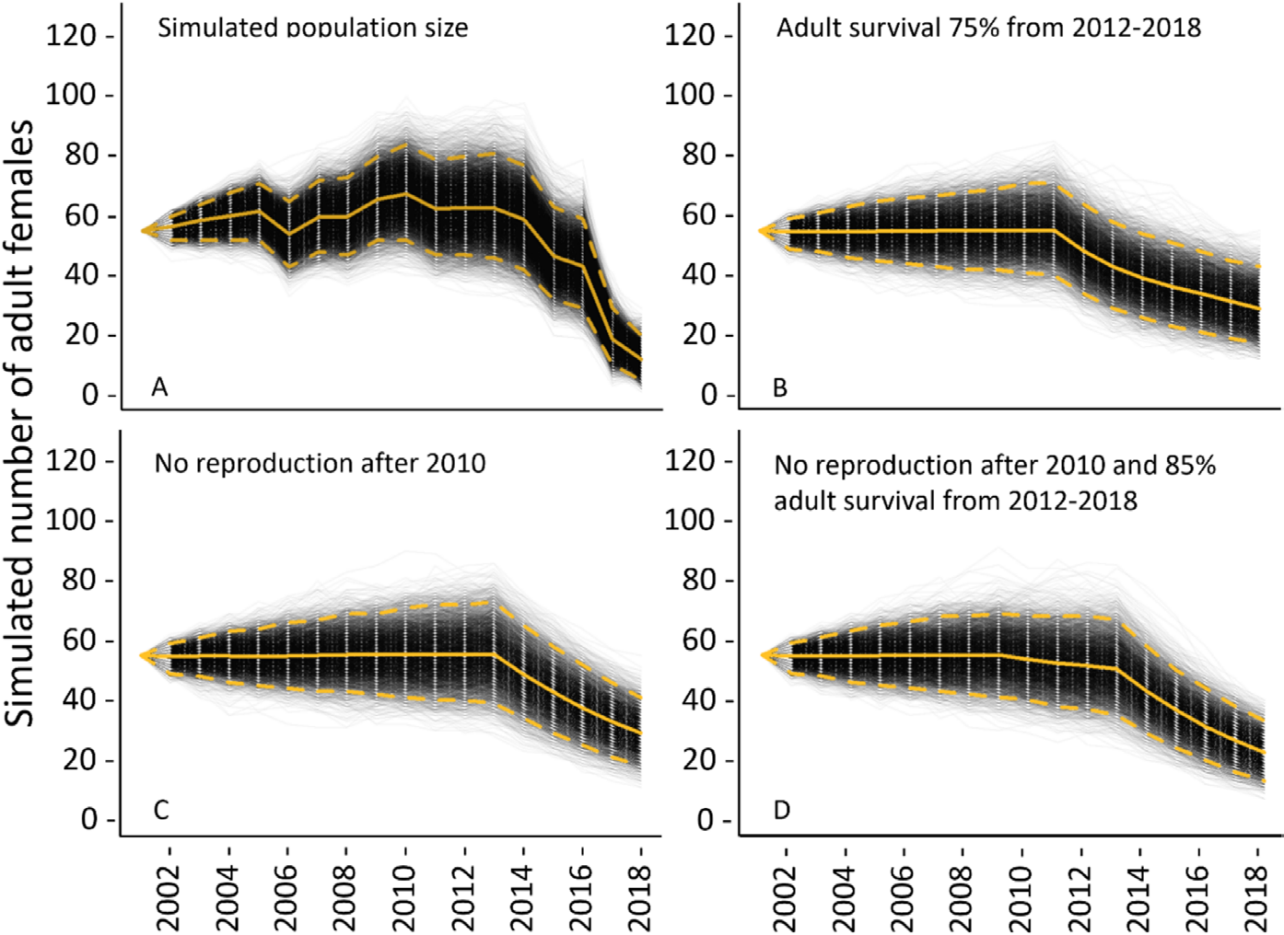
Simulated annual number of adult (≥ 4 years) female terrapins based on initial number of females captured during 2001 and estimated yearly survival from a CJS model (A) compared to three female population size scenarios (B–D) depicting low survival in different age classes (Table 1). Black lines represent each of 10,000 simulations, solid yellow line represents the mean across simulations, and dashed yellow lines indicate the 95% confidence limits (2.5% and 97.5% quantile). Population size scenarios (B–D) are similar to the annual population size estimates (A), where declines begin around 2014. The scenario in (B) represents a moderate degree of reduced adult survival (75%) annually from 2012 to 2018 (i.e., the years captures were lowest) and could be considered either permanent adult emigration out of the site or death (including from potential poaching). The scenario in (C) represents no recruitment into the population (i.e., no reproduction), from 2010 onward; this date was selected because our observed decline occurred around 2014, and 2010 accounts for the ~4‐year time lag it takes for turtles to mature. The scenario in (D) represents a combination of (B, C) where no reproduction occurs after 2010 and adult survival is reduced by 15% from 2012 to 2018, either because of permanent emigration or from death or harvesting.

Based on the initial number of female terrapins captured in 2001, annual apparent survival estimates, and Leslie matrix vital rates for adult females from a stable age distribution, we estimated an initial population size of 55 female terrapins (Figure 5A), corresponding to an initial total of 110 adult terrapins given an observed sex ratio of 50:50 at this study site. From 2002 to 2014, the estimated number of females moderately increased (e.g., 2003: mean 59, 95% CI 52–64; 2009: mean 66, 95% CI 52–80; 2010: mean 68, 95% CI 52–84; Figure 5A). Thereafter, the estimated number of females began to steadily decline (2015: mean 47, 95% CI 32–64; 2016: mean 43, 95% CI 29–60; 2017: mean 19, 95% CI 10–29), and dropped to 12 females in 2018 (95% CI 5–20; Figure 5A). These estimates are not meant to convey an exact population estimate, but rather, are intended to provide inference on the relative population changes over time (Figure 5).

### 3.6 Population Decline Simulation

Of 11 simulated annual abundance scenarios (Figure S1), three have a pattern similar to our estimated population size (Figure 5A). More specifically, these scenarios include (1) adult survival reduced to 75% from 2012 to 2018, (2) no reproduction after 2010, and (3) no reproduction after 2010 and 85% adult survival from 2012 to 2018 (Figure 5B–D). For each of these matching scenarios, the simulated number of adult (≥ 4 years old) female terrapins was consistently around 55–57 individuals from 2001 to 2011 (95% CI approximately 42–68; Figure 5B–D). Thereafter, declines begin, with the estimates falling to 27 (95% CI 18–41; Figure 5B), 28 (95% CI 19–40; Figure 5C), and 21 (95% CI 10–38) by 2018, across the three scenarios, respectively. Other simulated annual abundance scenarios incorporating effects of hurricanes on fecundity or survival of hatchlings and adults did not match the pattern of decline we observed (Figure S1B–E). Likewise, simulated scenarios of (1) 50% adult survival in 2010, (2) 60% and 85% adult survival from 2012 to 2018, and (3) no reproduction after 2010 coupled with 75% adult survival from 2012 to 2018 did not match the pattern of decline we observed (Figure S1F,H,I,K).

## 4 Discussion

Our study is the first to estimate adult survival rates for the mangrove diamond‐backed terrapin subspecies. We describe annual population changes for this species within ENP, over the course of 18 years, and document a steep population decline in this long‐lived species of conservation concern. Between 2001 and 2014, the number of captures (mean 117/year) and survival rates were relatively high (77%–96%), and during this time annual estimates of the number of adult females varied from 55 to 68 female terrapins (corresponding to a population size of ~110–136 adults, given a 1:1 sex ratio). However, captures began to decline between 2014 and2015, and we estimated a total of 12 female terrapins by 2018 (estimated population size of 24 terrapins). Return visits in 2020, 2021, and 2022 resulted in 0, 5, and 3 individuals observed, respectively. Simulated annual abundance scenarios show support for a combination of no reproduction occurring after 2010 and/or a 15%–25% annual reduction in adult survival from 2012 to 2018. We have considered several potential drivers for this apparent population decline and discuss these below.

### 4.1 Scenario A: Harvest

Current threats from illegal harvesting and overcollection for the pet trade are not well documented. In turtle populations, adults tend to have high annual survival rates, often above 90% (Wilbur and Morin 1988; Congdon and Gibbons 1990; Iverson 1991; Congdon et al. 1994; Heppell 1998). We explored the likelihood that the decline we observed was due to low adult survival, either because of emigration out of the study system, death, or removal of turtles by humans (i.e., illegal harvest). We generated scenarios of low adult survival (Figure S1), and three produced a pattern of decline that was similar to annual population size estimates (Figure 5A). One of these scenarios was a reduction in adult survival to 75% starting around 2012 that is sustained through 2018 (Figure 5B). In this scenario, 25% of adult terrapins leave the mangrove creek system each year, either through permanent emigration or harvest. Terrapins have high site fidelity to their creeks, remaining from year to year in the same tidal creek, with little or no interchange among the adult populations of adjoining creeks (Gibbons et al. 2001; Szerlag and McRobert 2006; Hart and McIvor 2008; Lamont et al. 2023). As well, female terrapins, who can make long excursions for nesting forays, typically return to the same creeks and marshes (Gibbons et al. 2001; Szerlag and McRobert 2006). Given that body condition index was stable over the course of the study and prey availability appeared consistent (Figure 2), it is unlikely that a systematic subset of adults permanently emigrated out of their creeks each year beginning in 2012. For the first half of our study, terrapins were abundant, and the nonturbid waters of the creek system make it easy to identify and collect individuals from a boat with a dipnet. Beginning in 2015, we initiated additional annual surveys of surrounding creek systems within 15 km to determine if terrapins from our study had moved to other creeks; none were found. Historical observations from researchers in 1998 documented large numbers of terrapins in Big Sable Creek, but none were ever observed in any neighboring creeks (Hardin Waddle, U.S. Geological Survey, Written Communication, 4/1/2024). Our study area is in a remote and relatively challenging location to access, within the vast coastline of ENP. As a result, it is difficult for law enforcement to monitor these areas. Rather than permanent emigration to other creeks, we suggest a more plausible scenario is a systematic removal of adult terrapins each year, beginning in 2012.

Overexploitation of wildlife for commercial trade is fueled by the growth of social media, which has generated widespread demand for exotic species and made international wildlife trade easier through rapid information exchange (Nekaris et al. 2013; Lavorgna 2014; Bennett et al. 2021). This extends to widespread illegal trading in threatened turtle species (Nijman and Shepherd 2007; Auliya et al. 2016; Sigouin et al. 2017). The United States has a rich assemblage of freshwater turtles (Mittermeier et al. 2015) and is one of the top turtle exporters (Hughes 1999; Ceballos and Fitzgerald 2004; Collis and Fenili 2011). Notably, with the shift to online commerce, the commercial trade of turtles across the globe now exhibits boom and bust cycles, such that a species (or location) is in high demand and heavily traded for a time, and then the market shifts to a new species or location (Klemens 2000; Van Dijk et al. 2000; CITES 2024).

Diamond‐backed terrapins are a vulnerable species that have sustained range‐wide population declines (Roosenburg et al. 2019) and are classified as a species that could be threatened with extinction unless trade is closely controlled (CITES 2024). The harvest or possession of terrapins is regulated in the 16 states where the species occurs, with 11 banning harvest, 7 allowing possession of a specific number of animals or with a permit, and 5 allowing aquaculture (i.e., farming) with a permit (Kennedy 2018). In 2022, Florida banned the harvest, take, possession, transport, or sale of terrapins or their eggs, except by permit (Florida Administrative Code 68A‐25.002).

Despite existing regulations, demand for diamond‐backed terrapins in the pet trade is strong (OPA 2019; Easter et al. 2023) and is likely facilitated by their ease of collection and colorful and ornate appearance, particularly the subspecies in our study, *M. t. rhizophorarum* (Roosenburg et al. 2019; Easter and Carter 2024). Commercial interest in turtles and terrapins remains high, primarily for trade and consumption in Asia or to Asian communities in North America, but also within the United States (NJAO 2015; FWC 2019; USDOJ 2021, 2023, 2024; Macdonald 2021; Fieseler 2021; USAO 2022; Easter et al. 2023). There have been many cases of overexploitation, illegal collection, smuggling, and selling of diamond‐backed terrapins. For example, 3522 wild adult diamond‐backed terrapins were harvested in New Jersey in 2013 and sold to an aquaculture facility in Maryland that exported over 14,000 hatchling terrapins to Asia the following year (NJAO 2015). In 2019, over 3500 diamond‐backed terrapins were trafficked in Pennsylvania (USDOJ 2018; OPA 2019). In Florida in 2019, a wildlife trafficking ring was intercepted while smuggling more than 4000 illegally captured turtles, including diamond‐backed terrapins, and selling them to large‐scale reptile dealers and illegal distributors who sold the turtles in Asia for between $300 and $10,000 (FWC 2019). Poachers targeted habitats known for specific species, depleted those areas, and then expanded to other parts of Florida to meet growing demands (FWC 2019).

### 4.2 Scenario B: No Reproduction After 2010

Another scenario of low adult survival which produced a pattern of decline similar to our annual population size estimates (Figure 5A) was one where there was no reproduction occurring after 2010 (Figure 5C). We focus this scenario on hatchling terrapins because adult female terrapins exhibit nest‐site fidelity within and across years (Gibbons et al. 2001; Szerlag‐Egger and McRobert 2007; Sheridan et al. 2010; Crawford et al. 2014) and despite weather events, shifting shorelines over decadal time frames, and a dynamic ecosystem, there continues to be an extensive network of sandy beaches along Cape Sable near our study creeks (Wanless and Vlaswinkel 2005). For this scenario, 2010 was chosen because capture numbers began to decline in 2014, and thus 2010 accounts for the approximate 4‐year time lag it takes for turtles to mature and potentially recruit into our study system. Little is known about terrapins in the time intervening from hatching and when they reach a carapace length of 8.0 cm, or three growing seasons (Baker et al. 2018). One interesting aspect of the structure of some terrapin populations is the absence of juvenile turtles, as has been documented in Delaware, South Carolina, and Florida populations (Hurd et al. 1979; Seigel 1984; Gibbons et al. 2001; Hart and McIvor 2008). The near total absence of juvenile (< 2 years old) terrapins in some populations is likely because they occupy habitats not typically sampled by researchers and are thought to be behaviorally cryptic, occurring in high marsh or upland areas for several years after hatching (Lovich et al. 1991; Gibbons et al. 2001; Muldoon and Burke 2012). Within our mangrove creek system, terrapins were adults, and as with Gibbons et al. (2001), the smallest females we found were approximately the same size as the smallest males (i.e., two subadults; a female, 9.0 cm SPL, and one male, 8.7 cm SPL). A lack of juveniles in our study may be a result of the mangrove creek open water matrix, which is not adjacent to upland habitat, which may present physiological challenges from prolonged submergence in a hypertonic environment or too much exposure to predators (reviewed in Baker et al. 2018). Further, because the nearest beaches suitable for nesting are 2.8–5 km from the mangrove creek network where adults reside, hatchlings may reside near the upland‐water interface for several years as they mature and disperse into the surrounding creek systems. Consequently, juveniles may not make their way into our creek system with regularity. It is possible that the population experienced reproductive failure from 2010 onward if maturing individuals stopped recruiting into our creek system. However, throughout the duration of the study, new (unmarked) recruits of older age classes (> 9.0 cm SCL) appeared in successive years. Specifically, recapture rates were 50%–60% from 2003 to 2013, with captures of approximately 120–150 individuals during each of these years, indicating that about half of captures were new individuals (Figure 1C). From 2013 to 2019, annual recapture rates were approximately 80%, indicating 20% of annual captures were new individuals (Figure 1C). A slowing rate of new recruits frequently occurs as a result of saturation, where most individuals in the population at a particular time point have become marked. While it seems possible that annual recruitment could be diluted by the time it takes hatchlings to mature and disperse into the mangrove creek network 2.8–5 km away, it does not seem as likely to be the sole driver of the decline, given that new individuals were regularly captured each year and because terrapins are a long‐lived species. This driver alone would also not explain our declining estimates of adult survival after 2014 from our CJS model. For wild terrapins, longevity is at least 20 years (Seigel 1984) and as much as 40 years (Hildebrand 1932). In this study, based on intervening years between capture, seven terrapins out of 656 were at least 20 years old. Another long‐term study in South Carolina has documented 31 terrapins over 25 years, with at least one individual 35 years old, based on mature size at capture (9.0 cm PL) and 30 intervening years between captures (K. Cecala, Sewanee University of the South, Written Communication, 4/25/2024). This long lifespan suggests that if the cause of the decline was related to recruitment, the population would slowly wane over many years, rather than exhibiting the abrupt decline we observed.

### 4.3 Scenario C: Combination of Harvest and No Reproduction

The third simulated annual abundance scenario with a pattern of decline similar to annual population estimates (Figure 5A) was a combination of reduced adult survival (85%) from 2012 to 2018 (Scenario A) and no reproduction after 2010 (Figure 5D). In this scenario, 15% of adult terrapins left the mangrove creek system each year (i.e., permanently emigrating, dying, or by human removal), and in addition, no recruitment was observed after 2010. In addition to Scenario A, it is plausible that some reproductive failure was occurring because of maturing individuals not recruiting back into the creek system; however, our CJS‐based estimates of adult survival dropped well below 50% for 2017–2018.

### 4.4 Prey Availability

We have considered several potential drivers for this apparent population decline. One less likely cause is a change in prey availability. The stable relationship between terrapin mass and body length over the course of the study does not support this potential driver (Figure 2). In other words, for a given body length, from 1 year to the next, terrapins did not lose mass, indicating a stable prey base. A previous study at this same location examined the terrapin diet in 2012 and identified a wide array of prey from fecal samples comprising bivalves (*n* = 3 genera), gastropods (*n* = 6 genera), crustaceans (fiddler, tree, and mud crabs, isopods), barnacles, and fish (Denton et al. 2016). As with terrapins in other parts of their range, terrapins in the Everglades are generalists, feeding on the resources available in the environment (Denton et al. 2016). Although we did not quantify the available prey base throughout the study, we did not observe a notable shift in the prey base, as snails (e.g., *Littorina angulifera* , *Melampus coffeus* ), bivalves, barnacles, and crabs (fiddler, tree, mud) appeared prevalent throughout the duration of our study. Subsequent terrapin fecal samples collected in 2016, 2018, and 2019 contained these same or similar species (Mathew Denton, U.S. Geological Survey, Written Communication 6/24/2024).

### 4.5 Trapping

In other areas of their range, major contributing factors to terrapin population declines are crab pots and road mortality, contributing to unbalanced sex ratios not observed in this study. For example, commercial and recreational use of crab pots from Texas to New Jersey attract large numbers of terrapins into traps where they drown as bycatch (Bishop 1983; Roosenburg et al. 1997; Hoyle and Gibbons 2000; Roosenburg and Green 2000; Dorcas et al. 2007; Grosse et al. 2011). Whereas crab pots disproportionately kill small terrapins, particularly males that do not outgrow the gape limitation of commercial wire crab pots (e.g., Dorcas et al. 2007), road mortality contributes to significant declines in the number of adult females (Wood and Herlands 1997; Szerlag and McRobert 2006; Avissar 2006). However, ENP is a vast wilderness and there are no roads near our terrapin population, nor is crab trapping permitted in the park. Across the 98 surveys we have conducted in our mangrove creek system, we have not observed any evidence of crab trapping occurring (e.g., crab pots, buoys, terrapin carcasses).

### 4.6 Habitat Loss and Hurricanes

Habitat loss, particularly from the development of nesting beaches, has been identified as a threat to terrapin populations (reviewed in Maerz et al. 2018). While there has been no anthropogenic destruction of the habitat in our mangrove creeks, the Big Sable Creek area of Cape Sable has been affected by five hurricanes since the 1900s: the Labor Day Storm (1935), Donna (1960), Andrew (1992), Wilma (2005), and Irma (2017; Smith et al. 2009; Castañeda‐Moya et al. 2010; Wingard et al. 2020). Hurricanes Wilma and Irma coincided with the duration of our study. Hurricane Wilma made landfall on October 24, 2005, as a category 3 storm, approximately 80 km northwest of our study area (Pasch et al. 2006) and the storm surge was as much as ~3.0 m in Big Sable Creek (Smith et al. 2009). Hurricane Irma made landfall on September 10, 2017, as a category 4 storm approximately 76 km southwest of our study area (Cangialosi et al. 2021). Hurricanes have played a significant role in shaping the Greater Everglades ecosystem of south Florida, with both destructive (e.g., damage to coastal mangroves, conversion to mudflats) and constructive effects (e.g., nutrient and sediment deposition; Perkins and Enos 1968; Risi et al. 1995; Smith et al. 2009; Whelan et al. 2009; Castañeda‐Moya et al. 2010; Wingard et al. 2020). Based on marginally lower survival estimates in hurricane years (Figure 3), we explored the likelihood that hurricane occurrence was responsible for the terrapin decline we observed. Hurricane scenarios were meant to simulate years when nests may have flooded, nesting beaches lost, or hatchlings died or were displaced from the study area; in these cases, there may have been no survival for young age classes. Similarly, there may have been low (50%) adult survival in those years. However, none of our simulations incorporating low hatchling or adult survival during hurricane years produced a pattern of decline matching annual estimates (Figure S1). It is estimated that south Florida has been struck by 40 hurricanes between 1871 and 2003, with an average frequency of about one per 3 years (Lodge 2016). Mangrove forests are considered highly resilient to tropical storms and hurricanes (Castañeda‐Moya et al. 2010) but also are inherently dynamic ecosystems, exposed to regular disturbance (e.g., storm surges; Smith et al. 2009). Terrapins throughout their range, including south Florida, have evolved in these dynamic coastal ecosystems and can be resilient to hurricane disturbance (e.g., Denton et al. 2023). Turtles, including terrapins, can remain submerged or buried in mud for long periods of time through aquatic respiration or metabolic regulation (Mccutcheon 1943; Jackson and Ultsch 2010; Williard and Harden 2011). It is likely that terrapins either buried themselves in sediment prior to hurricane landfall, or if displaced by currents or winds, returned to the area after storm surges receded. In subsequent years after Hurricane Wilma, we continued to observe and capture individuals caught prior to Hurricane Wilma in 2005 (Figure 1B). For example, in 2006 we captured 150 terrapins (female, *n* = 68; male, *n* = 82) and 64% were recaptured (Figure 1C). Previous studies have documented terrapins remaining in or returning to their creeks after hurricanes (Mealey et al. 2014; Denton et al. 2023; Lamont et al. 2023). Further, while we are not aware of locations that marked terrapins in our study have nested, there are several undisturbed (i.e., minimal human impacts) beaches within 2.8–5 km of Big Sable Creek that appear to be suitable for terrapins given well‐documented nesting use by American crocodiles (Mazzotti et al. 2022) and sea turtles (Davis and Whiting 1977). Based on historic imagery taken every 3 years from 1985 to 2023 (Google Earth 2024) these beaches have remained intact, and we have observed terrapins on them. Therefore, although survival estimates were marginally lower during hurricane years (Figure 3), based on our observations and in conjunction with hurricane population simulations, these disturbances are not responsible for the terrapin decline at Big Sable Creek.

### 4.7 Disease, Predation, and Water Quality

Terrapins, especially eggs and hatchlings, are vulnerable to terrestrial predators, including raccoons, foxes, crabs, shorebirds, rats, and ants (reviewed in Maerz et al. 2018). However, terrestrial mortality from mesomammals, such as raccoons, tends to be higher in urban areas where they are subsidized, or because nesting beaches are concentrated as a result of development (Maerz et al. 2018). Our study occurs in a remote area of a national park with no urban development. Concomitant with this study, we conducted extensive evening surveys for crocodiles and nesting marine turtles during the past two decades on the beaches of Cape Sable, which are the nearest nesting area for terrapins. Although anecdotal and not quantified, we have not observed increases in raccoon sightings during the past two decades. Further, multiple studies have documented severe mammal declines in ENP, attributed to predation by invasive Burmese pythons, a species that does not consume turtles (reviewed in Guzy et al. 2023). For example, the frequency of mammal observations (raccoons, opossums, bobcats, rabbits, gray foxes, and white‐tailed deer) declined by 85%–100% from 2003 to 2011 (Dorcas et al. 2012). Taken together, it appears unlikely that increased predation rates could be the cause of the terrapin decline in this study.

Other considerations for what could have caused the decline we observed are potential water quality changes or disease. We have not observed outward indications of diseases in terrapins during our study (e.g., no lesions, tumors, signs of respiratory infection, reduced body condition index, or carcasses). Salinity has varied over the course of our study, with 2009–2010 characterized as a period of coastal drought and high salinity, and then later, from 2012 to 2013, salinity was low because of high rainfall in the Cape Sable region, after which time salinity increased from 2014 to 2016, and has since become more cyclical annually (USGS 2024). Salinity could be expected to influence primary production and subsequently terrapin prey that consume vegetation. However, given that the terrapin body condition index remained stable over the course of the study, it appears likely that salinity did not influence terrapin survival.

## 5 Challenges With Interpretation

We cannot be sure what caused the decline we observed. Detecting and measuring population declines is challenging because populations are dynamic and fluctuate (e.g., Lawton 1994; Hanski 1998; McCain et al. 2016), and this can cause a lag prior to detecting changes. Studying population dynamics over time is labor intensive, expensive, and often logistically challenging, particularly when sites are remote and difficult to access, as in our study. Several factors of this study make interpreting the apparent decline challenging, including the remoteness of the population, which we selected to provide data on demography in an undisturbed population (e.g., no roads or crab trap pressure, Wood and Herlands 1997; Dorcas et al. 2007). However, as a result, the remoteness made surveys designed to estimate temporary emigration too labor intensive (i.e., robust design, Pollock 1982). In addition, ideal study sites should contain the entire population or the ability to sample the entire population. As with other populations (Hurd et al. 1979; Seigel 1984; Gibbons et al. 2001), our study only sampled adults, and there are few direct or indirect estimates of hatchling or juvenile annual survival (reviewed in Maerz et al. 2018). Overall, data on terrapin vital rates are sparse, and we used data from northern populations to parameterize much of our matrix population model (i.e., Mitro 2003) except for clutch size and clutches per season (Seigel 1980, 1984); to our knowledge, there were no published vital rates for southern Florida until this study. A lack of these regional vital rates may influence how accurate our estimates of annual population size may be, although the relative change in annual estimates is expected to be fairly accurate. Finally, another consideration for interpreting an apparent decline is the importance of site selection. Many natural populations exhibit long‐term population cycles, and for logistical reasons, researchers are more likely to conduct studies where the focal organism is abundant; studies are thus more likely to begin near a population's peak than a trough, and as a result, a time series may be more likely to show a decline (Fournier et al. 2019).

## 6 Conclusions

We documented the virtual disappearance of diamond‐backed terrapins from a mangrove creek complex in a remote region of ENP. Terrapins exhibit a characteristic life history of delayed reproduction, low nest and juvenile survival, and long lifespan. As such, high adult mortality can result in a rapid population decline and potentially local population extinctions (Congdon et al. 1993, 1994; Gibbons et al. 2001; Butler and Roosenburg 2018). Despite challenges with interpretation, we were able to identify some scenarios which were more plausible than others to describe causes for this decline. Based on our simulations, it seems unlikely that drops in recruitment alone were responsible for observed declines, and a sudden decrease in adult apparent survival—supported by both mark‐recapture analysis and simulation—appears to be an important driver of this decline. We have not observed terrapins in the surrounding creek networks; therefore, extirpation of adult terrapins in this mangrove creek ecosystem could create a void that could take a generation or more of recruitment to fill. However, if terrapins have been illegally removed, this population is unlikely to recover. This can have implications for species survival given the endemic and range‐restricted status of this subspecies, *M. t. rhizophorarum*, in southern Florida (Lovich and Hart 2018), where significant data gaps remain in their basic natural history. Our work here presented the first estimates of adult female survival, and the population projection matrices we constructed can be built upon in future work. Conservation remains a key objective throughout the terrapin's range, and information on hatchling and juvenile survival could be useful to identify management needs for these life stages. Results of this study may help inform threats to mangrove diamond‐backed terrapins, including the potential for illegal harvest, and can help identify protections needed for this species.

## Author Contributions

**Jacquelyn C. Guzy:** conceptualization (equal), data curation (lead), formal analysis (lead), investigation (equal), methodology (equal), supervision (lead), validation (lead), visualization (lead), writing – original draft (lead), writing – review and editing (lead). **Brian J. Smith:** conceptualization (equal), data curation (lead), formal analysis (lead), investigation (equal), methodology (equal), validation (lead), visualization (lead), writing – review and editing (equal). **Mathew J. Denton:** conceptualization (equal), data curation (equal), investigation (equal), methodology (equal). project administration (equal), writing – review and editing (equal). **Michael S. Cherkiss:** conceptualization (equal), investigation (equal), methodology (equal), project administration (equal), supervision (equal), writing – review and editing (equal). **David C. Roche:** conceptualization (equal), investigation (equal), methodology (equal). **Andrew G. Crowder:** conceptualization (equal), investigation (equal), methodology (equal). **Kristen M. Hart:** conceptualization (equal), funding acquisition (lead), investigation (equal), methodology (equal), project administration (lead), supervision (lead), writing – review and editing (equal).

## Ethics Statement

All terrapin captures and handling were performed under the following Everglades National Park permits: EVER‐2002‐SCI‐0092, EVER‐2004‐SCI‐0035, EVER‐2007‐SCI‐0031, EVER‐2009‐SCI‐0025, EVER‐2011‐SCI‐0036, EVER‐2013‐SCI‐0060, EVER‐2015‐SCI‐0068, EVER‐2017‐SCI‐0061, EVER‐2020‐SCI‐0006, EVER‐2021‐SCI‐0020, EVER‐2022‐SCI‐0008 and USGS Institutional Animal Care Protocol permits: USGS‐SESC‐IACUC‐2011‐05, USGS‐SESC‐2013‐04, USGS‐SESC‐2014‐02, USGS‐WARC‐GNV‐2017‐04, USGS‐WARC‐GNV‐2019‐14.

## Conflicts of Interest

The authors declare no conflicts of interest.

## Supporting information


**Figure S1.** Simulated annual number of adult (≥ 4 years) female terrapins based on initial number of females captured during 2001 and estimated yearly survival from a CJS model (A) compared to 11 female population size scenarios (B‐L) depicting low survival in different age classes (see Table 1). Black lines represent 10,000 simulations, solid yellow line represents the mean across simulations, and dashed yellow lines are the 95% confidence limits (2.5% and 97.5% quantile). Panels G, J, and L most closely match annual population size estimates (Figure 5A) and scenarios in remaining panels do not.

## Data Availability

Data and survival estimation and population size simulation code used in this manuscript are available at Guzy et al. (2025).

## References

[ece371347-bib-0001] Auliya, M. , S. Altherr , D. Ariano‐Sanchez , et al. 2016. “Trade in Live Reptiles, Its Impact on Wild Populations, and the Role of the European Market.” Biological Conservation 204: 103–119.

[ece371347-bib-0002] Avissar, N. G. 2006. “Changes in Population Structure of Diamondback Terrapins (*Malaclemys terrapin terrapin*) in a Previously Surveyed Creek in Southern New Jersey.” Chelonian Conservation and Biology 5: 154–159.

[ece371347-bib-0003] Baker, P. J. , R. E. J. Boerner , and R. C. Wood . 2018. “Hatchling Behavior and Overwintering.” In Ecology and Conservation of the Diamond‐Backed Terrapin, edited by W. M. Roosenburg and V. S. Kennedy , 93–110. John Hopkins University Press.

[ece371347-bib-0004] Bennett, E. L. , F. M. Underwood , and E. J. Milner‐Gulland . 2021. “To Trade or Not to Trade? Using Bayesian Belief Networks to Assess How to Manage Commercial Wildlife Trade in a Complex World.” Frontiers in Ecology and Evolution 9: 1–16. 10.3389/fevo.2021.587896.

[ece371347-bib-0005] Bishop, J. M. 1983. “Incidental Capture of Diamondback Terrapin by Crab Pots.” Estuaries 6, no. 4: 426–430. 10.2307/1351402.

[ece371347-bib-0006] Buhlmann, K. A. , T. S. B. Akre , J. B. Iverson , et al. 2009. “A Global Analysis of Tortoise and Freshwater Turtle Distributions With Identification of Priority Conservation Areas.” Chelonian Conservation and Biology 8: 116–149.

[ece371347-bib-0007] Buhlmann, K. A. , and T. D. Tuberville . 1998. “Use of Passive Integrated Transponder (PIT) Tags for Marking Small Freshwater Turtles.” Chelonian Conservation and Biology 3: 102–104.

[ece371347-bib-0008] Butler, J. , and W. Roosenburg . 2018. “The Future for Diamond‐Backed Terrapins.” In Ecology and Conservation of the Diamond‐Backed Terrapin, edited by W. M. Roosenburg and V. S. Kennedy , 265–268. John Hopkins University Press.

[ece371347-bib-0009] Butler, J. A. , C. Broadhurst , M. Green , and Z. Mullin . 2004. “Nesting, Nest Predation and Hatchling Emergence of the Carolina Diamondback Terrapin, *Malaclemys terrapin centrata* , in Northeastern Florida.” American Midland Naturalist 152: 145–155.

[ece371347-bib-0010] Butler, J. A. , G. L. Heinrich , and R. A. Siegel . 2006. “Third Workshop on the Ecology, Status, and Conservation of Diamondback Terrapins (*Malaclemys terrapin*): Results and Recommendations.” Chelonian Conservation and Biology 5, no. 2: 331–334. 10.2744/1071-8443(2006)5[331:TWOTES]2.0.CO;2.

[ece371347-bib-0011] Cagle, F. R. 1939. “A System of Marking Turtles for Future Identification.” Copeia 1939: 170–173.

[ece371347-bib-0012] Cangialosi, J. P. , A. S. Latto , and R. Berg . 2021. National Hurricane Center Tropical Cyclone Report: Hurricane Irma (AL112017).

[ece371347-bib-0013] Carr, A. 1952. Handbook of Turtles: The Turtles of the United States, Canada, and Baja California. Cornell University Press.

[ece371347-bib-0014] Castañeda‐Moya, E. , R. R. Twilley , V. H. Rivera‐Monroy , K. Zhang , S. E. Davis III , and M. Ross . 2010. “Sediment and Nutrient Deposition Associated With Hurricane Wilma in Mangroves of the Florida Coastal Everglades.” Estuaries and Coasts 33: 45–58.

[ece371347-bib-0015] Caswell, H. 2001. Matrix Population Models. 2nd ed. Sinauer.

[ece371347-bib-0016] Ceballos, C. P. , and L. A. Fitzgerald . 2004. “The Trade in Native and Exotic Turtles in Texas.” Wildlife Society Bulletin 32: 881–891.

[ece371347-bib-0017] Chambers, R. M. , and J. C. Maerz . 2018. “Bycatch in Blue Crab Fisheries.” In Ecology and Conservation of the Diamond‐Backed Terrapin, edited by W. M. Roosenburg and V. S. Kennedy , 231–244. John Hopkins University Press.

[ece371347-bib-0018] CITES . 2024. “Convention on International Trade in Endangered Species of Wild Fauna and Flora: Trade Statistics, Appendices I, II and III.” https://cites.org/eng/app/appendices.php.

[ece371347-bib-0019] Collis, A. H. , and R. N. Fenili . 2011. The Modern U.S. Reptile Industry Georgetown Economic Services LLC, Economic Analysis Group. Report ID 1018_04182011‐3.

[ece371347-bib-0020] Congdon, J. D. , A. Dunham , and R. C. V. L. Sels . 1993. “Delayed Sexual Maturity and Demographics of Blanding's Turtles (*Emydoidea blandingii*) Implications for Conservation and Management of Long‐Lived Organisms.” Conservation Biology 7, no. 4: 826–833. 10.1046/j.1523-1739.1993.740826.x.

[ece371347-bib-0021] Congdon, J. D. , A. E. Dunham , R. C. Van , and L. Sels . 1994. “Demographics of Common Snapping Turtles (*Chelydra serpentina*): Implications for Conservation and Management of Long‐Lived Organisms.” American Zoologist 34, no. 3: 397–408. 10.1093/icb/34.3.397.

[ece371347-bib-0022] Congdon, J. D. , and J. W. Gibbons . 1990. “The Evolution of Turtle Life Histories.” In Life History and Ecology of the Slider Turtle, edited by J. W. Gibbons . Smithsonian Institution Press.

[ece371347-bib-0023] Converse, P. E. , and S. R. Kuchta . 2018. “Molecular Ecology and Phylogeography.” In Ecology and Conservation of the Diamond‐Backed Terrapin, edited by W. M. Roosenburg and V. S. Kennedy , 51. John Hopkins University Press.

[ece371347-bib-0024] Cormack, R. M. 1964. “Estimates of Survival From the Sighting of Marked Animals.” Biometrika 51: 429–438.

[ece371347-bib-0025] Crawford, B. A. , J. C. Maerz , N. P. Nibbelink , K. A. Buhlmann , T. M. Norton , and S. E. Albeke . 2014. “Hot Spots and Hot Moments of Diamondback Terrapin Road‐Crossing Activity.” Journal of Applied Ecology 51: 367–375.

[ece371347-bib-0026] Davis, G. E. , and M. C. Whiting . 1977. “Loggerhead Sea Turtle Nesting in Everglades National Park, Florida, USA.” Herpetelogica 33: 18–28.

[ece371347-bib-0027] Denton, M. J. , K. M. Hart , A. W. J. Demopoulos , A. Oleinik , and J. D. Baldwin . 2016. “Diet of Diamondback Terrapins ( *Malaclemys terrapin* ) in Subtropical Mangrove Habitats in South Florida.” Chelonian Conservation and Biology 15: 54–61.

[ece371347-bib-0028] Denton, M. J. , K. M. Hart , J. Wnek , S. A. Moss , and H. W. Avery . 2023. “Isotopic Niche of New Jersey Terrapins Suggests Intraspecific Resource Partitioning, and Little Variability Following a Major Hurricane.” Hydrobiologia 850: 2975–2990.

[ece371347-bib-0029] Didham, R. K. , Y. Basset , C. M. Collins , et al. 2020. “Interpreting Insect Declines: Seven Challenges and a Way Forward.” Insect Conservation and Diversity 13: 103–114.

[ece371347-bib-0030] Doak, D. , P. Kareiva , and B. Klepetka . 1994. “Modeling Population Viability for the Desert Tortoise in the Western Mojave Desert.” Ecological Applications 4: 446–460.

[ece371347-bib-0031] Doak, D. F. , J. A. Estes , B. S. Halpern , et al. 2008. “Understanding and Predicting Ecological Dynamics: Are Major Surprises Inevitable?” Ecology 89: 952–961. 18481520 10.1890/07-0965.1

[ece371347-bib-0133] Donini, J. , C. Lechowicz , and R. A. Valverde . 2018. “Comparisons of Summer and Winter Patterns in Ovarian Development, Plasma Vitellogenin, and Sex Steroids in Female Diamondback Terrapins (Malaclemys terrapin) in Southern Florida.” Chelonian Conservation and Biology 17: 227–235.

[ece371347-bib-0032] Dorcas, M. E. , and J. W. Gibbons . 2013. “Long‐Term Ecological Research on America's Only Estuarine Turtle: The Diamondback Terrapin.” In Reptiles in Research: Investigations of Ecology, Physiology, and Behavior From Desert to Sea, edited by W. I. Lutterschmidt , 447–461. Nova Biomedical.

[ece371347-bib-0033] Dorcas, M. E. , J. D. Willson , and J. W. Gibbons . 2007. “Crab Trapping Causes Population Decline and Demographic Changes in Diamondback Terrapins Over Two Decades.” Biological Conservation 137: 334–340.

[ece371347-bib-0136] Dorcas, M. E. , J. D. Willson , R. N. Reed , et al. 2012. “Severe Mammal Declines Coincide with Proliferation of Invasive Burmese Pythons in Everglades National Park.” Proceedings of the National Academy of Sciences of the United States of America 109, no. 7: 2418–2422. 22308381 10.1073/pnas.1115226109 PMC3289325

[ece371347-bib-0034] Drabeck, D. H. , M. W. H. Chatfield , and C. L. Richards‐Zawacki . 2014. “The Status of Louisiana's Diamondback Terrapin (*Malaclemys terrapin*) Populations in the Wake of the Deepwater Horizon Oil Spill: Insights From Population Genetic and Contaminant Analyses.” Journal of Herpetology 48, no. 1: 125–136. 10.1670/12-186.

[ece371347-bib-0035] Easter, T. , and N. Carter . 2024. “Analysis of 20 Years of Turtle Exports From the US Reveals Mixed Effects of CITES and a Need for Better Monitoring.” Conservation Science and Practice: e13092.

[ece371347-bib-0036] Easter, T. , J. Trautmann , M. Gore , and N. Carter . 2023. “Media Portrayal of the Illegal Trade in Wildlife: The Case of Turtles in the US and Implications for Conservation.” People and Nature 5: 758–773. 10.1002/pan3.10448.

[ece371347-bib-0037] Ernst, C. H. , and J. E. Lovich . 2009. Turtles of the United States and Canada. Johns Hopkins University Press.

[ece371347-bib-0038] Feinberg, J. A. , and R. L. Burke . 2003. “Nesting Ecology and Predation of Diamondback Terrapins, *Malaclemys terrapin* , at Gateway National Recreation Area, New York.” Journal of Herpetology 37: 517–526.

[ece371347-bib-0039] Fieseler, C. 2021. “To Catch a Turtle Thief: Blowing the Lid off an International Smuggling Operation.” Walrus. https://thewalrus.ca/to‐catch‐a‐turtle‐thief/.

[ece371347-bib-0040] Fournier, A. M. V. , E. R. White , and S. B. Heard . 2019. “Site‐Selection Bias and Apparent Population Declines in Long‐Term Studies.” Conservation Biology 33: 1370–1379. 31210365 10.1111/cobi.13371

[ece371347-bib-0041] FWC . 2019. “Florida Fish and Wildlife Conservation Commission. FWC Busts Wildlife Trafficking Ring Smuggling Thousands of Turtles, Returns Turtles to Wild.” https://content.govdelivery.com/accounts/FLFFWCC/bulletins/266e905.

[ece371347-bib-0042] Gibbons, J. W. , J. E. Lovich , A. D. Tucker , N. N. FitzSimmons , and J. L. Greene . 2001. “Demographic and Ecological Factors Affecting Conservation and Management of the Diamondback Terrapin (*Malaclemys terrapin*) in South Carolina.” Chelonian Conservation and Biology 4: 66–74.

[ece371347-bib-0043] Google Earth . 2024. “Cape Sable, Florida.” http://www.google.com/earth/index.html.

[ece371347-bib-0044] Grosse, A. M. , J. C. Maerz , J. Hepinstall‐Cymerman , and M. E. Dorcas . 2011. “Effects of Roads and Crabbing Pressures on Diamondback Terrapin Populations in Coastal Georgia.” Journal of Wildlife Management 75, no. 4: 762–770. 10.1002/jwmg.104.

[ece371347-bib-0045] Guzy, J. C. , M. J. Denton , M. S. Cherkiss , B. J. Smith , D. C. Roche , and K. M. Hart . 2025. “Capture Histories and Body Size Data for a Population of Mangrove Diamond‐Backed Terrapins (*Malaclemys terrapin rhizophorarum*) Everglades National Park, Florida, USA, 2001‐2019: U.S. Geological Survey Data Release.” 10.5066/P13UKAUV.

[ece371347-bib-0046] Guzy, J. C. , B. G. Falk , B. J. Smith , et al. 2023. “Burmese Pythons in Florida: A Synthesis of Biology, Impacts, and Management Tools.” NeoBiota 80: 1–119.

[ece371347-bib-0047] Hanski, I. 1998. “Metapopulation Dynamics.” Nature 396: 41–49.

[ece371347-bib-1999] Hart, K. M. 2005. Population Biology of Diamondback Terrapins (Malaclemys terrapin): Defining and Reducing Threats Across Their Geographic Range. Duke University.

[ece371347-bib-0048] Hart, K. M. , M. E. Hunter , T. L. King , et al. 2014. “Regional Differentiation Among Populations of the Diamondback Terrapin (*Malaclemys terrapin*).” Conservation Genetics 15, no. 3: 593–603. 10.1007/s10592-014-0563-6.

[ece371347-bib-0049] Hart, K. M. , and C. C. McIvor . 2008. “Demography and Ecology of Mangrove Diamondback Terrapins in a Wilderness Area of Everglades National Park, Florida, USA.” Copeia 2008, no. 1: 200–208. 10.1643/CE-06-161.

[ece371347-bib-0050] Hauswaldt, J. S. , and T. C. Glenn . 2005. “Population Genetics of the Diamondback Terrapin ( *Malaclemys terrapin* ).” Molecular Ecology 14: 723–732. 15723664 10.1111/j.1365-294X.2005.02451.x

[ece371347-bib-0051] Heppell, S. S. 1998. “Application of Life‐History Theory and Population Model Analysis to Turtle Conservation.” Copeia 1998: 367–375.

[ece371347-bib-0052] Heppell, S. S. , L. B. Crowder , and D. T. Crouse . 1996. “Models to Evaluate Headstarting as a Management Tool for Long‐Lived Turtles.” Ecological Applications 6: 556–565.

[ece371347-bib-0053] Heppell, S. S. , L. B. Crowder , and T. R. Menzel . 1999. “Life Table Analysis of Long‐Lived Marine Species With Implications for Conservation and Management.” American Fisheries Society Symposium 23: 137–148.

[ece371347-bib-0054] Hildebrand, S. F. 1932. “Growth of Diamond‐Back Terrapins Size Attained, Sex Ratio and Longevity.” Zoologica 9: 551–563.

[ece371347-bib-0055] Hoyle, M. E. , and J. W. Gibbons . 2000. “Use of a Marked Population of Diamondback Terrapins (*Malaclemys terrapin*) to Determine Impacts of Recreational Crab Pots.” Chelonian Conservation and Biology 3: 735–737.

[ece371347-bib-0056] Hughes, B. B. , R. Beas‐Luna , A. K. Barner , et al. 2017. “Long‐Term Studies Contribute Disproportionately to Ecology and Policy.” Bioscience 67: 271–281.

[ece371347-bib-0057] Hughes, D. W. 1999. “The Contribution of the Pet Turtle Industry to the Louisiana Economy.” Aquaculture Economics and Management 3: 205–214.

[ece371347-bib-0058] Hurd, I. , G. Smedes , and T. Dean . 1979. “An Ecological Study of a Natural Population of Diamondback Terrapins (*Malaclemys t. terrapin*) in a Delaware.” Estuaries 2: 28–33.

[ece371347-bib-0059] Iverson, J. B. 1991. “Patterns of Survivorship in Turtles (Order Testudines).” Canadian Journal of Zoology 69: 385–391.

[ece371347-bib-0060] Jackson, D. C. , and G. R. Ultsch . 2010. “Physiology of Hibernation Under the Ice by Turtles and Frogs.” Journal of Experimental Zoology Part A: Ecological Genetics and Physiology 313A: 311–327. 10.1002/jez.603 20535765

[ece371347-bib-0061] Jolly, G. M. 1965. “Explicit Estimates From Capture‐Recapture Data With Both Death and Immigration‐Stochastic Model.” Biometrika 52: 225–247. 14341276

[ece371347-bib-0062] Kennedy, V. S. 2018. “History of Commercial Fisheries and Artificial Propagation.” In Ecology and Conservation of the Diamond‐Backed Terrapin, edited by W. M. Roosenburg and V. S. Kennedy , 187–197. Johns Hopkins University Press.

[ece371347-bib-0063] Klemens, M. W. 2000. Turtle Conservation. Smithsonian Institution Press.

[ece371347-bib-0064] Laake, J. , and E. Rexstad . 2022. “RMark‐An Alternative Approach to Building Linear Models in MARK. Page Program MARK– A ‘Gentle Introduction’.”

[ece371347-bib-0065] Laake, J. L. 2013. “RMark: An R Interface for Analysis of Capture‐Recapture Data With MARK.”

[ece371347-bib-0066] Lamont, M. M. , M. E. Price , and D. J. Catizone . 2023. “Satellite Telemetry Reveals Space Use of Diamondback Terrapins.” Animal Biotelemetry 11, no. 1: 42. 10.1186/s40317-023-00354-x.

[ece371347-bib-0067] Lavorgna, A. 2014. “Wildlife Trafficking in the Internet Age.” Crime Science 3: 5.

[ece371347-bib-0068] Lawton, J. H. 1994. “Population Dynamic Principles.” Philosophical Transactions of the Royal Society of London. Series B: Biological Sciences 344: 61–68.

[ece371347-bib-0070] Lindenmayer, D. B. , G. E. Likens , C. J. Krebs , and R. J. Hobbs . 2010. “Improved Probability of Detection of Ecological “Surprises”.” Proceedings of the National Academy of Sciences of the United States of America 107, no. 51: 21957–21962. 10.1073/pnas.1015696107. 21098660 PMC3009814

[ece371347-bib-0071] Lodge, T. E. 2016. “The Everglades Handbook: Understanding the Ecosystem.” In Page the Everglades Handbook: Understanding the Ecosystem, 4th ed. CRC Press.

[ece371347-bib-0072] Lovett, G. M. , D. A. Burns , C. T. Driscoll , et al. 2007. “Who Needs Environmental Monitoring?” Frontiers in Ecology and the Environment 5: 253–260.

[ece371347-bib-0073] Lovich, J. E. , J. R. Ennen , M. Agha , and J. W. Gibbons . 2018a. “Where Have all the Turtles Gone, and Why Does It Matter?” Bioscience 68: 771–781.

[ece371347-bib-0074] Lovich, J. E. , J. W. Gibbons , and K. M. Greene . 2018b. “Life History With Emphasis on Geographic Variation.” In Ecology and Conservation of the Diamond‐Backed Terrapin, edited by W. E. Roosenburg and V. S. Kennedy , 277. John Hopkins University Press.

[ece371347-bib-0075] Lovich, J. E. , and J. W. Gibbons . 1990. “Age at Maturity Influences Adult Sex Ratio in the Turtle *Malaclemys terrapin* .” Oikos 59, no. 1: 126–134. 10.2307/3545132.

[ece371347-bib-0076] Lovich, J. E. , and K. M. Hart . 2018. “Taxonomy: A History of Controversy and Uncertainty.” In Ecology and Conservation of the Diamond‐Backed Terrapin, edited by W. E. Roosenburg and V. S. Kennedy , 37–50. John Hopkins University Press.

[ece371347-bib-0077] Lovich, J. E. , A. D. Tucker , D. E. Kling , J. W. Gibbons , and T. D. Zimmerman . 1991. Behavior of Hatchling Diamondback Terrapins Released in a South Carolina Salt Marsh.

[ece371347-bib-0121] Macdonald, B . 2021. “U.S. Fish & Wildlife Service: International Turtle‐Trafficking Ring Busted.” https://www.fws.gov/story/2021‐10/international‐turtle‐trafficking‐ring‐busted.

[ece371347-bib-0078] Maerz, J. C. , R. A. Seigel , and B. A. Crawford . 2018. “Conservation in Terrestrial Habitats: Mitigating Habitat Loss, Road Mortality, and Subsidized Predators.” In Ecology and Conservation of the Diamond‐Backed Terrapin, edited by W. E. Roosenburg and V. S. Kennedy , 201–220. John Hopkins University Press.

[ece371347-bib-0079] Magnuson, J. J. 1990. “Long‐Term Ecological Research and the Invisible Present.” Bioscience 40: 495–501.

[ece371347-bib-0080] Mazzotti, F. J. , S. A. Balaguera‐Reina , L. A. Brandt , et al. 2022. “Natural and Anthropogenic Factors Influencing Nesting Ecology of the American Crocodile in Florida, United States.” Frontiers in Ecology and Evolution 10: 1–14. 10.3389/fevo.2022.904576.

[ece371347-bib-0081] McCain, C. , T. Szewczyk , and K. B. Knight . 2016. “Population Variability Complicates the Accurate Detection of Climate Change Responses.” Global Change Biology 22, no. 6: 2081–2093. 10.1111/gcb.13211. 26725404

[ece371347-bib-0082] McCutcheon, F. H. 1943. “The Respiratory Mechanism in Turtles.” Ecological and Evolutionary Physiology 16: 255–269.

[ece371347-bib-0083] Mealey, B. K. , J. D. Baldwin , G. B. Parks‐Mealey , G. D. Bossart , and M. R. J. Forstner . 2014. “Characteristics of Mangrove Diamondback Terrapins ( *Malaclemys terrapin rhizophorarum* ) Inhabiting Altered and Natural Mangrove Islands.” Journal of North American Herpetology 2014: 76–80.

[ece371347-bib-0084] Meyer, C. F. J. , L. M. S. Aguiar , L. F. Aguirre , et al. 2010. “Long‐Term Monitoring of Tropical Bats for Anthropogenic Impact Assessment: Gauging the Statistical Power to Detect Population Change.” Biological Conservation 143, no. 11: 2797–2807. 10.1016/j.biocon.2010.07.029.

[ece371347-bib-0085] Mitro, M. G. 2003. “Demography and Viability Analyses of a Diamondback Terrapin Population.” Canadian Journal of Zoology 81: 716–726.

[ece371347-bib-0086] Mittermeier, R. A. , P. P. Van Dijk , A. G. J. Rhodin , and S. D. Nash . 2015. “Turtle Hotspots: An Analysis of the Occurrence of Tortoises and Freshwater Turtles in Biodiversity Hotspots, High‐Biodiversity Wilderness Areas, and Turtle Priority Areas.” Chelonian Conservation and Biology 14: 2–10.

[ece371347-bib-0087] Montevecchi, W. A. , and J. Burger . 1975. “Aspects of the Reproductive Biology of the Northern Diamondback Terrapin *Malaclemys terrapin terrapin* .” American Midland Naturalist 94, no. 1: 166–178. 10.2307/2424547.

[ece371347-bib-0088] Muldoon, K. A. , and R. L. Burke . 2012. “Movements, Overwintering, and Mortality of Hatchling Diamond‐Backed Terrapins ( *Malaclemys terrapin* ) at Jamaica Bay, New York.” Canadian Journal of Zoology 90: 651–662.

[ece371347-bib-0089] Nekaris, B. K. A. I. , N. Campbell , T. G. Coggins , E. J. Rode , and V. Nijman . 2013. “Tickled to Death: Analysing Public Perceptions of “Cute” Videos of Threatened Species (Slow Lorises—*Nycticebus* spp.) on Web 2.0 Sites.” PLoS One 8: 1–10. 10.1371/journal.pone.0069215. PMC3722300 23894432

[ece371347-bib-0090] Nijman, V. , and C. R. Shepherd . 2007. “Trade in Non‐Native, CITES‐Listed, Wildlife in Asia, as Exemplified by the Trade in Freshwater Turtles and Tortoises (*Chelonidae*) in Thailand.” Contributions to Zoology 76: 207–211.

[ece371347-bib-0091] NJAO . 2015. “New Jersey Administrative Order No. 2015‐02.” Accessed April 19. http://www.nj.gov/dep/docs/ao2015‐02.pdf.

[ece371347-bib-0092] OPA . 2019. “Office of Public Affairs, United States Department of Justice.” Pennsylvania Man Sentenced for Trafficking Protected Turtles. https://www.justice.gov/opa/pr/pennsylvania‐man‐sentenced‐trafficking‐protected‐turtles.

[ece371347-bib-0093] Ost, M. , S. Ramula , A. Linden , P. Karell , and M. Kilpi . 2016. “Small‐Scale Spatial and Temporal Variation in the Demographic Processes Underlying the Large‐Scale Decline of Eiders in the Baltic Sea.” Population Ecology 58: 121–133.

[ece371347-bib-0094] Pasch, R. J. , E. S. Blake , H. D. I. Cobb , and D. P. Roberts . 2006. Tropical Cyclone Report Hurricane Wilma.

[ece371347-bib-0095] Perkins, R. D. , and P. Enos . 1968. “Hurricane Betsy in the Florida‐Bahama Area‐ Geologic Effects and Comparison With Hurricane Donna.” Journal of Geology 76: 710–717.

[ece371347-bib-0096] Pollock, K. H. 1982. “A Capture‐Recapture Design Robust to Unequal Probability of Capture.” Journal of Wildlife Management 46, no. 3: 752–757. 10.2307/3808568.

[ece371347-bib-0097] R Core Team . 2023. R: A Language and Environment for Statistical Computing. R Foundation for Statistical Computing. https://www.R‐project.org/.

[ece371347-bib-0098] Rhodin, A. G. J. , C. B. Stanford , P. P. Van Dijk , et al. 2018. “Global Conservation Status of Turtles and Tortoises (Order Testudines).” Chelonian Conservation and Biology 17, no. 2: 135–161. 10.2744/CCB-1348.1.

[ece371347-bib-0099] Risi, A. , H. R. Wanless , L. P. Tedesco , and S. Gelsanliter . 1995. “Catastrophic Sedimentation From Hurricane Andrew Along the Southwest Florida Coast.” Journal of Coastal Research, no. 21: 83–102. https://www.jstor.org/stable/25736002.

[ece371347-bib-0100] Roosenburg, W. M. 1990. “Final Report: Chesapeake Diamondback Terrapin Investigations for the Period 1987, 1988, and 1989. Solomons, MD.”

[ece371347-bib-0101] Roosenburg, W. M. , P. J. Baker , R. Burke , M. E. Dorcas , and R. C. Wood . 2019. *Malaclemys terrapin*, the IUCN Red List of Threatened Species.

[ece371347-bib-0102] Roosenburg, W. M. , W. Cresko , M. Modesitte , and M. B. Robbins . 1997. “Diamondback Terrapin ( *Malaclemys terrapin* ) Mortality in Crab Pots.” Conservation Biology 11: 1166–1172.

[ece371347-bib-0132] Roosenburg, W. M. , and A. E. Dunham . 1997. “Allocation of Reproductive Output: Egg–and Clutch‐Size Variation in the Diamondback Terrapin.” Copeia 1997: 290–297.

[ece371347-bib-0103] Roosenburg, W. M. , and J. P. Green . 2000. “Impact of a Bycatch Reduction Device on Diamondback Terrapin and Blue Crab Capture in Crab Pots.” Ecological Applications 10: 882–889.

[ece371347-bib-0104] Roosenburg, W. M. , and V. S. Kennedy . 2018. “Ecology and Conservation of the Diamond‐Backed Terrapin.” In Ecology and Conservation of the Diamond‐Backed Terrapin, edited by W. E. Roosenburg and V. S. Kennedy . John Hopkins University Press.

[ece371347-bib-0105] Seber, G. A. F. 1965. “A Note on the Multiple‐Recapture Census.” Biometrika 52: 249–259. 14341277

[ece371347-bib-0106] Seigel, R. A. 1980. “Nesting Habits of Diamondback Terrapins (*Malaclemys terrapin*) on the Atlantic Coast of Florida.” Transactions of the Kansas Academy of Science 83, no. 4: 239–246. 10.2307/3628414.

[ece371347-bib-0107] Seigel, R. A. 1984. Parameters of Two Populations of Diamondback Terrapins ( *Malaclemys terrapin* ) on the Atlantic Coast of Florida. Vertebrate Ecology and Systematics—A Tribute to Henry S. Fitch, 77–87. University of Kansas Museum of Natural History.

[ece371347-bib-0108] Seigel, R. A. , R. B. Smith , J. Demuth , L. M. Ehrhart , and F. F. Snelson . 2002. “Amphibians and Reptiles of the John F. Kennedy Space Center, Florida: A Long‐Term Assessment of a Large Protected Habitat (1975‐2000).” Florida Scientist 65: 1–12.

[ece371347-bib-0109] Sheridan, C. M. , J. R. Spotila , W. F. Bien , and H. W. Avery . 2010. “Sex‐Biased Dispersal and Natal Philopatry in the Diamondback Terrapin, *Malaclemys terrapin* .” Molecular Ecology 19: 5497–5510. 21091556 10.1111/j.1365-294X.2010.04876.x

[ece371347-bib-0110] Sigouin, A. , M. Pinedo‐Vasquez , R. Nasi , C. Poole , B. Horne , and T. M. Lee . 2017. “Priorities for the Trade of Less Charismatic Freshwater Turtle and Tortoise Species.” Journal of Applied Ecology 54: 345–350.

[ece371347-bib-0111] Smith, T. J. I. , G. H. Anderson , K. Balentine , G. Tiling , G. A. Ward , and K. R. T. Whelan . 2009. “Cumulative Impacts of Hurricanes on Florida Mangrove Ecosystems: Sediment Deposition, Storm Surges and Vegetation.” Wetlands 29, no. 1: 24–34. 10.1672/08-40.1.

[ece371347-bib-0112] Stanford, C. B. , J. B. Iverson , A. G. J. Rhodin , et al. 2020. “Turtles and Tortoises Are in Trouble.” Current Biology 30, no. 12: R721–R735. 10.1016/j.cub.2020.04.088. 32574638

[ece371347-bib-0113] Stubben, C. , and B. Milligan . 2007. “Estimating and Analyzing Demographic Models Using the Popbio Package in R.” Journal of Statistical Software 22 no. 11: 1–23. 10.18637/jss.v022.i11.

[ece371347-bib-0114] Szerlag, S. , and S. P. McRobert . 2006. “Road Occurrence and Mortality of the Northern Diamondback Terrapin.” Applied Herpetology 3: 27–37.

[ece371347-bib-0115] Szerlag‐Egger, S. , and S. P. McRobert . 2007. “Northern Diamondback Terrapin Occurrence, Movement, and Nesting Activity Along a Salt Marsh Access Road.” Chelonian Conservation and Biology 6, no. 2: 295–301. 10.2744/1071-8443(2007)6[295:NDTOMA]2.0.CO;2.

[ece371347-bib-0116] USAO . 2022. “U.S. Attorney's Office, Southern District of Florida: Broward Wildlife Dealer and Company Sentenced in Scheme to Harvest and Sell Florida Turtles.” https://www.justice.gov/usao‐sdfl/pr/broward‐wildlife‐dealer‐and‐company‐sentenced‐scheme‐harvest‐and‐sell‐florida‐turtles.

[ece371347-bib-0117] USDOJ . 2018. “United States Department of Justice, Eastern District of Pennsylvania. Levittown Man Indicted for Trafficking Protected Turtles.” https://www.justice.gov/usao‐edpa/pr/levittown‐man‐indicted‐trafficking‐protected‐turtles.

[ece371347-bib-0118] USDOJ . 2021. “United States Department of Justice, Environmental Crimes Bulletin September 2021. United States v. Yuan Xie et al., Nos. 6:19‐CR‐00503, 6:20‐CR00218.” https://www.justice.gov/enrd/blog/ecs‐bulletin‐october‐2021.

[ece371347-bib-0119] USDOJ . 2023. “United States Department of Justice. Florida Man Who Trafficked in Florida Box Turtles, Loggerhead Musk Turtles, and Ornate Diamondback Terrapins Pleads Guilty to Lacey Act Violations.” https://www.justice.gov/usao‐mdfl/pr/florida‐man‐who‐trafficked‐florida‐box‐turtles‐loggerhead‐musk‐turtles‐and‐ornate.

[ece371347-bib-0120] USDOJ . 2024. “United States Department of Justice. Lee County, Florida Man Sentenced to Prison for Conspiring to Smuggle Turtles to Germany and Hong Kong and Falsely Labeling the Turtles on Related Paperwork.” https://www.justice.gov/usao‐sdfl/pr/lee‐county‐florida‐man‐sentenced‐prison‐conspiring‐smuggle‐turtles‐germany‐and‐hong.

[ece371347-bib-0122] USGS . 2024. “U.S. Geological Survey Coastal Salinity Index.” https://apps.usgs.gov/sawsc/csi/index.html.

[ece371347-bib-0123] Van Dijk, P. P. , B. L. Stuart , and A. G. J. Rhodin . 2000. “Asian Turtle Trade: Proceedings of a Workshop on Conservation and Trade of Freshwater Turtles and Tortoises in Asia. Chelonian Research Monographs:85.”

[ece371347-bib-0124] Wanless, H. R. , and B. M. Vlaswinkel . 2005. “Coastal Landscape and Channel Evolution Affecting Critical Habitats at Cape Sable, Everglades National Park, Miami, FL.”

[ece371347-bib-0125] Watts, K. , R. C. Whytock , K. J. Park , et al. 2020. “Ecological Time Lags and the Journey Towards Conservation Success.” Nature Ecology & Evolution 4: 304–311. 31988448 10.1038/s41559-019-1087-8

[ece371347-bib-0126] Whelan, K. R. T. , T. J. Smith , G. H. Anderson , and M. L. Ouellette . 2009. “Hurricane Wilma's Impact on Overall Soil Elevation and Zones in a Mangrove Forest.” Wetlands 29: 16–23.

[ece371347-bib-0127] Wilbur, H. M. , and P. J. Morin . 1988. “Life History Evolution in Turtles.” In Biology of the Reptilia, edited by C. Gans and R. B. Huey , 387–439. Alan R. Liss, Inc.

[ece371347-bib-0128] Williard, A. S. , and L. A. Harden . 2011. “Seasonal Changes in Thermal Environment and Metabolic Enzyme Activity in the Diamondback Terrapin (*Malaclemys terrapin*).” Comparative Biochemistry and Physiology. Part A, Molecular & Integrative Physiology 158: 477–484. 10.1016/j.cbpa.2010.12.005. 21147242

[ece371347-bib-0129] Wingard, G. L. , S. E. Bergstresser , B. L. Stackhouse , et al. 2020. “Impacts of Hurricane Irma on Florida bay Islands, Everglades National Park, USA.” Estuaries and Coasts 43: 1070–1089.

[ece371347-bib-0130] Wood, R. C. 1997. “The Impact of Commercial Crab Traps on Northern Diamondback Terrapins, *Malaclemys terrapin terrapin* .” In Proceedings: Conservation, Restoration, and Management of Tortoises and Turtles‐An International Conference, 21–27. New York Turtle and Tortoise Society.

[ece371347-bib-0131] Wood, R. C. , and R. Herlands . 1997. “Turtles and Tires: The Impact of Roadkills on Northern Diamondback Terrapin, *Malaclemys terrapin terrapin*, Populations on the Cape May Peninsula, Southern New Jersey, USA.” In Proceedings: Conservation, Restoration, and Management of Tortoises and Turtles—An International Conference, 46–53. New York Turtle and Tortoise Society.

[ece371347-bib-0134] White, E. R. 2019. “Minimum time required to detect population trends: the need for long‐term monitoring programs.” BioScience 69, no. 1: 40–46.

