# Supplementary material for: Interpreting a Sudden Population Decline in a Long‐Lived Species ( Malaclemys terrapin rhizophorarum )
Source: Ecol Evol. 2025 May 7;15(5):e71347. doi: 10.1002/ece3.71347 (PMC12058646; doi:10.1002/ece3.71347)
Supplement: Supplementary file 1 — Figure S1. Simulated annual number of adult (≥ 4 years) female terrapins based on initial number of females captured during 2001 and estimated yearly survival from a CJS model (A) compared to 11 female population size scenarios (B‐L) depicting low survival in different age classes (see Table 1). Black lines represent 10,000 simulations, solid yellow line represents the mean across simulations, and dashed yellow lines are the 95% confidence limits (2.5% and 97.5% quantile). Panels G, J, and L most closely match annual population size estimates (Figure 5A) and scenarios in remaining panels do not. [file ECE3-15-e71347-s001.docx]

#
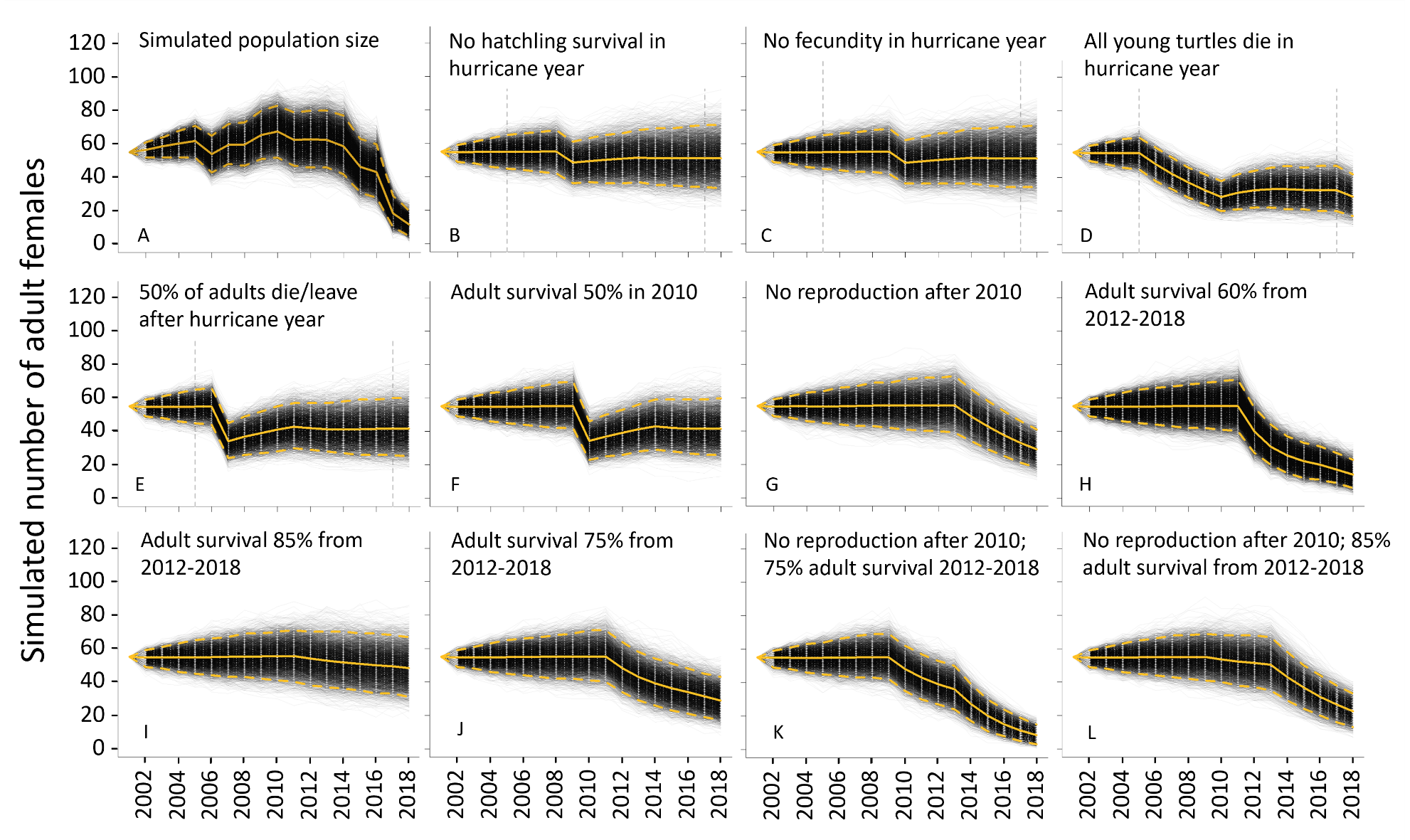
Supplemental Figure 1

Supplemental Figure 1: Simulated annual number of adult (≥ 4 years) female terrapins based on initial number of females captured during 2001 and estimated yearly survival from a CJS model (A) compared to 11 female population size scenarios (B-L) depicting low survival in different age classes (see Table 1). Black lines represent 10,000 simulations, solid yellow line represents the mean across simulations, and dashed yellow lines are the 95% confidence limits (2.5% and 97.5% quantile). Panels G, J, and L most closely match annual population size estimates (Fig. 5A) and scenarios in remaining panels do not.
